# The *Dendrobium catenatum* Lindl. genome sequence provides insights into polysaccharide synthase, floral development and adaptive evolution

**DOI:** 10.1038/srep19029

**Published:** 2016-01-12

**Authors:** Guo-Qiang Zhang, Qing Xu, Chao Bian, Wen-Chieh Tsai, Chuan-Ming Yeh, Ke-Wei Liu, Kouki Yoshida, Liang-Sheng Zhang, Song-Bin Chang, Fei Chen, Yu Shi, Yong-Yu Su, Yong-Qiang Zhang, Li-Jun Chen, Yayi Yin, Min Lin, Huixia Huang, Hua Deng, Zhi-Wen Wang, Shi-Lin Zhu, Xiang Zhao, Cao Deng, Shan-Ce Niu, Jie Huang, Meina Wang, Guo-Hui Liu, Hai-Jun Yang, Xin-Ju Xiao, Yu-Yun Hsiao, Wan-Lin Wu, You-Yi Chen, Nobutaka Mitsuda, Masaru Ohme-Takagi, Yi-Bo Luo, Yves Van de Peer, Zhong-Jian Liu

**Affiliations:** 1 Shenzhen Key Laboratory for Orchid Conservation and Utilization, The National Orchid Conservation Center of China and The Orchid Conservation and Research Center of Shenzhen, Shenzhen 518114, China; 2 State Key Laboratory of Systematic and Evolutionary Botany, Institute of Botany, Chinese Academy of Sciences, Beijing 100093, China; 3 Shenzhen Key Lab of Marine Genomics, State Key Laboratory of Agricultural Genomics, Shenzhen 518083, China; 4 Dapartment of Life Sciences, National Cheng Kung University, Tainan 701, Taiwan; 5 Orchid Research Center, National Cheng Kung University, Tainan 701, Taiwan; 6 Institute of Tropical Plant Sciences, National Cheng Kung University, Tainan 701, Taiwan; 7 Graduate School of Science and Engineering, Saitama University, Saitama 338-8570, Japan; 8 The Center for Biotechnology and BioMedicine, Graduate School at Shenzhen, Tsinghua University, Shenzhen 518055, China; 9 Technology Center, Taisei Corporation, Kanagawa 245-0051, Japan; 10 Haixia Institute of Science and Technology, Fujian Agriculture and Forestry University, Fuzhou 350002, China; 11 Fruit Crop Systems Biology Laboratory, College of Horticulture, Nanjing Agricultural University, Nanjing 210095, China; 12 College of Forestry, South China Agricultural University, Guangzhou, 510640, China; 13 Chinese Academy of Forestry, Beijing, 100093, China; 14 PubBio-Tech Services Corporation, Wuhan 430070, China; 15 Bioproduction Research Institute, National Institute of Advanced Industrial Science and Technology, Ibaraki 305-8562, Japan; 16 Department of Plant Systems Biology, VIB, and Department of Plant Biotechnology and Bioinformatics. Ghent University, Ghent, Belgium; 17 Bioinformatics Institute Ghent, Ghent University, Ghent B-9000, Belgium; 18 Department of Genetics, Genomics Research Institute, Pretoria, South Africa

## Abstract

Orchids make up about 10% of all seed plant species, have great economical value, and are of specific scientific interest because of their renowned flowers and ecological adaptations. Here, we report the first draft genome sequence of a lithophytic orchid, *Dendrobium catenatum.* We predict 28,910 protein-coding genes, and find evidence of a whole genome duplication shared with Phalaenopsis. We observed the expansion of many resistance-related genes, suggesting a powerful immune system responsible for adaptation to a wide range of ecological niches. We also discovered extensive duplication of genes involved in glucomannan synthase activities, likely related to the synthesis of medicinal polysaccharides. Expansion of MADS-box gene clades *ANR1*, *StMADS11,* and MIKC^*^, involved in the regulation of development and growth, suggests that these expansions are associated with the astonishing diversity of plant architecture in the genus *Dendrobium*. On the contrary, members of the type I MADS box gene family are missing, which might explain the loss of the endospermous seed. The findings reported here will be important for future studies into polysaccharide synthesis, adaptations to diverse environments and flower architecture of Orchidaceae.

Orchids, constituting approximately 10% of all seed plant species, have enormous value for commercial horticulture, and are of specific scientific interest because of their spectacular flowers, ecological adaptations^1^,^2^ and secondary metabolites^3^,^4^,^5^,^6^. *Dendrobium* is the third largest genus of Orchidaceae and contains approximately 1,450 species, characterised by a fleshy stem with abundant polysaccharides and growing in diverse habitats^3^,^4^,^5^,^6^. A draft genome sequence of *Dendrobium officinale* Kimura & Migo has been reported before but the highly fragmented assembly and the presence of multiple peaks in *K*-mer analyses, suggesting that its sequence is likely derived from an artificial hybrid^7^, seriously complicate correct interpretation of the genome. To complement the lack of a high quality, well assembled genome sequence for *Dendrobium*, we here present the genome of *D. catenatum* Lindl., a lithophytic orchid found in subtropical and temperate regions^2^ and commonly used as a health food in many Asian countries^3^,^4^,^5^. Analysis of the *D. catenatum* genome sequence offers insights into flower development and polysaccharide synthesis, as well as its wide distribution.

## Results

### Genome sequencing and genome characteristics

*Dendrobium catenatum* (Supplementary Note 1) has thirty-eight (2N = 2X = 38) small chromosomes of approximately 2 μm (Supplementary Note 2 and Supplementary Fig. 1). To sequence its complete genome, we generated a total of 222.51 Gb of raw reads, with multiple insert libraries ranging in size from 180 bp to 20 Kb (Supplementary Table 1). A *K*-mer analysis estimated the genome size of *D. catenatum* at 1.11 Gb (Supplementary Fig. 2). Assembly was done with SOAPdenovo2^8^ and Platanus^9^, but completeness and N50 length of scaffolds were much better with the latter tool (Supplementary Fig. 3), the results of which were used in subsequent analyses. The total length of its assembly was 1.01 Gb (Supplementary Table 2). Mapping all of the paired-end reads to the assembly revealed that 97% of the sequence had a coverage depth greater than five (Supplementary Fig. 4). Further quality analysis indicated that 93% of the set of eukaryotic core genes (CEGMA)^10^ were present and 97% were partially represented, suggesting near completeness of the euchromatin component. In addition, 93%–95% of the RNA seq data set could be mapped onto the assembled sequence (Supplementary Tables 3 and 4). These results suggest that our genome assembly is of high quality.

**Figure 1:**
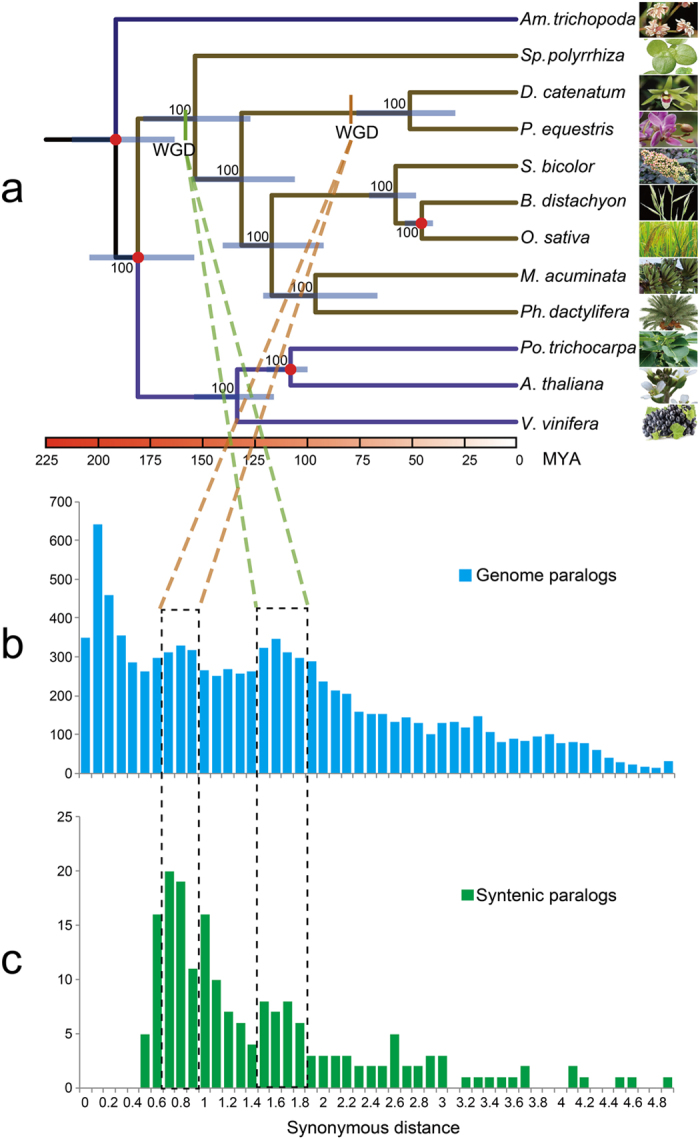
Phylogenetic position and Ks distributions for *D. catenatum*. (**a**) Phylogenetic tree showing the topology and divergence times for 12 plant species, including *D. catenatum*. Estimated divergence times are indicated by light blue boxes at internodes. Numbers at nodes indicate bootstrap values. The brown bar indicates the orchid-specific whole-genome duplication (WGD), while the green bar indicates a more ancient monocot-specific WGD (Thanks Li-Jun Chen for taking the images of species). (**b**) Distribution of synonymous substitutions per synonymous site (Ks) for the whole *D. catenatum* paranome. (**c**) Distribution of synonymous substitutions per synonymous site (Ks) for orthologous genes found in syntenic regions. Two consistent peaks highlighted by the dashed rectangles are considered to reflect the most recent and older WGD events.

A total of 789 Mb of repetitive elements occupying more than 78.1% of the *D. catenatum* genome were annotated using a method combining structural and homology information. Retrotransposable elements, known to be the dominant form of repeats in angiosperm genomes, constituted a large part of the *D. catenatum* genome and included the most abundant subtypes, such as LTR/Copia (27.36%), LTR/Gypsy (18.49%), LINE/L1 (8.44%) and LINE/RTE (5.68%), among others. In addition, the percentage of *de novo* predicted repeats was notably larger than that obtained for repeats based on Repbase^11^, indicating that *D. catenatum* has many unique repeats compared to other sequenced plant genomes (Supplementary Note 3 and Supplementary Table 5). Among these elements, long terminal repeats (LTRs) were the most dominant type, accounting for approximately 46% of the genome. After calculating their times of insertion, we discovered that a burst of LTR activity occurred during the last five million years (Supplementary Fig. 5) and therefore, we deduced that these LTRs were inserted into the genome after *D. catenatum* diverged from *Phalaenopsis* species (which is estimated to have occurred 22.6–59.6 million years ago, Fig. 1). We annotated 28,910 protein-coding genes (Supplementary Note 4), of which 22,394 (74.9%) were supported by transcriptome data (Supplementary Fig. 6 and Supplementary Table 6). Notably, we found that *D. catenatum* has, on average, longer genes than most other sequenced plant species, although similar to that of the butterfly orchid *Phalaenopsis equestris* (Shauer) Rchb. f.^12^, due to both species having longer average intron lengths (Supplementary Fig. 7 and Supplementary Table 7). Therefore, this feature might be a unique characteristic of Orchidaceae. In addition, we identified 49 microRNAs, 310 transfer RNAs, 248 ribosomal RNAs and 144 small nuclear RNAs in the *D. catenatum* genome (Supplementary Table 8).

We determined the expansion and contraction of orthologous protein families among species using CAFÉ2.2^13^, which is based on a probabilistic graphical model. For each species, expanded and contracted (compared with their ancestors) gene families were compared with *D. catenatum* to identify gene families that were uniquely expanded or contracted in *D. catenatum* (Supplementary Note 5). Seven hundred and fifty-six gene families were found to be expanded in *D. catenatum* (30 of these significantly) and 804 families contracted (of which four significantly; Supplementary Fig. 8). For the significantly expanded gene families, we conducted GO enrichment analysis and found enrichment for the GO terms ‘DNA metabolic process’, ‘cellular macromolecule metabolic process’, ‘RNA-directed DNA polymerase activity’, ‘primary metabolic process’ and ‘ribonuclease H activity’ (Supplementary Table 9).

We identified 5,758,781 heterozygous single nucleotide polymorphisms (SNPs) in the *D. catenatum* genome. The heterozygous SNP rate for the whole genome was estimated at 6.28 × 10^–3^, whereas the SNP rate in exons was as low as 4.98 × 10^–3^ (Supplementary Fig. 9). Of the 139,830 SNPs that were found in exons, 69,459 caused non-synonymous mutations, affecting 18,404 genes, and this suggested that *D. catenatum* is a high heterozygosity genome. We conducted a Gene Ontology (GO)^14^ and KEGG^15^ enrichment analyses of the affected genes and found enrichment of the KEGG pathways ‘Biosynthesis of secondary metabolites’, ‘Plant hormone signal transduction’, ‘Metabolic pathways’ and ‘Isoflavonoid biosynthesis’(Supplementary Table 10), and the GO terms ‘ATP binding’, ‘protein tyrosine kinase activity’ and ‘transition metal ion binding’ (Supplementary Table 11).

### Genome evolution

We constructed a highly supported phylogenetic tree and estimated the divergence times of 12 plants based on genes extracted from a total of 677 single-copy families (Fig. 1a). As expected, we found that the *D. catenatum* is most closely related to *P. equestris* from which it separated approximately 38 million years ago.

Both the distribution of synonymous substitutions per synonymous site (Ks) across all paralogous genes (regardless of gene order, Fig. 1b) and for duplicated genes lying in synteny blocks (Fig. 1c) show two obvious peaks at Ks values between 0.7–0.9 and 1.5–1.8, suggestive of two rounds of whole-genome duplication (WGDs) in the *D. catenatum* lineage (Supplementary Note 6). Dating of the WGDs suggests that the most recent WGD appeared near to the Cretaceous–Paleogene (K/Pg) boundary^16^ and is shared with the WGD event documented recently for *P. equestris*^12^. Since it has been suggested that WGDs might facilitate species diversification^17^,^18^, it would be interesting to see whether the WGD has also been shared with the species-rich subfamily Orchidoideae (3630 species), which diverged from the Epidendroideae (about 20,000 species, amongst which *D. catenatum* and *P. equestris*) about 59 million years ago^19^, and with the subfamilies Apostasioideae, Vanilloideae and Cypripedioideae, which only include 17, 185 and 180 species, respectively^20^. Cypripedioideae and the ancestor of Orchidoideae and Epidendroideae subfamilies are assumed to have diverged from each other about 68 million years ago^19^. Unfortunately, whole genome sequences, or extensive transcriptome data sets from members of these other subfamilies are not yet available. The older peak in the Ks age distribution probably points to one or more older WGD events that have occurred in the monocot lineage, as already previously suggested^21^.

### Gene family evolution

We have also zoomed in on some specific gene families.

#### Terpene synthase genes

**Figure 2:**
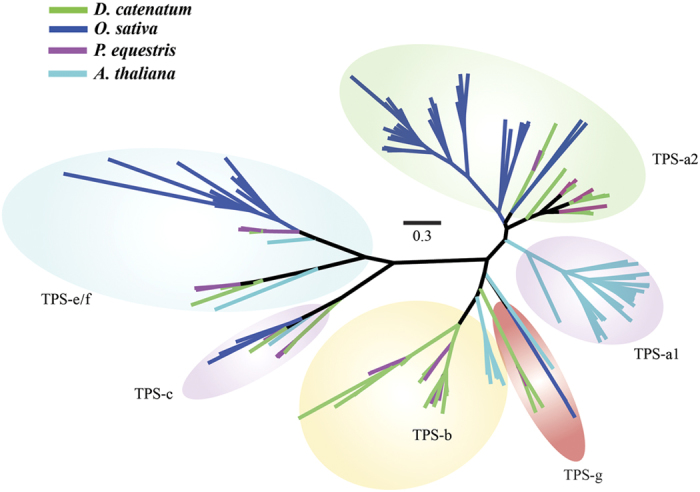
Phylogeny of putative full-length TPSs from *D. catenatum* (green), *P. equestris* (purple), *O. sativa* (blue) and *A. thaliana* (cyan). See text for details.

As secondary metabolites, most plant terpenes and their corresponding synthases have evolved selectively to increase fitness by adaptation to specific ecological niches^22^. Plant terpene synthase (*TPS*) genes can be divided into seven subfamilies (*a, b, c, d, e/f, g* and *h*)^22^. The *TPS* genes of *D. catenatum* and *P. equestris* all fall into known angiosperm *TPS* clades, *TPS-a*, *TPS-b*, *TPS-e/f*, *TPS-c*, and *TPS-g* (Fig. 2). The genome of *D. catenatum* encodes 39 members of *TPS*, whereas there are only 21 in the genome of *P. equestris.* Notably, rapid expansion by tandem gene duplication is particularly common in the *TPS*-a subfamily of these two orchids. Furthermore, the specific placement of *TPS-a* genes for *D. catenatum* and *P. equestris* (Fig. 2; Supplementary Tables 12 and 13) implies that the expansion of this gene family has occurred in the ancestor of the Epidendroideae subfamily, or at least prior to the divergence of *D. catenatum* and *P. equestris*, and might have contributed to species radiation in this subfamily, containing over 20,000 species. Indeed, although this needs to be further investigated, a previous study has suggested that the expansion of the *TPS-a* subfamily might be linked to the radiation of the flowering plants^23^.

#### Disease resistance genes

Plant disease resistance genes (*R* genes) play a key role in recognizing proteins expressed by specific avirulence genes of pathogens^21^, and form various subfamilies, such as the TIR-domain-containing (for example, TOLL/INTERLEUKIN LIKE RECEPTOR/RESISTANCE PROTEIN) (TIR-NB-LRR), the non-TIR-domain containing (NB-LRR), and the non-TIR coiled-coil domain-containing (CC-NB-LRR) R-protein subfamilies^24^. The genomes of *D. catenatum* and *P. equestris* possess 157 and 79 *R* genes, respectively (Supplementary Table 14). Although further investigation is required, the dramatic expansion of its *R* genes suggests that *D. catenatum* may possess a more powerful disease immune system than *P. equestris*.

#### Heat-shock proteins

As molecular chaperones, heat-shock proteins (Hsp) are ubiquitous in plant cells. Hsp genes are not only associated with stress caused by heat shock and other abiotic factors, but have recently also been found to be associated with response to biotic stress^25^,^26^. Hsp genes function to manage the stress-induced denaturation of other proteins and can be classified into seven major families based on their molecular weight: small Hsps, Hsp20, Hsp40, Hsp60, Hsp70, Hsp90 and Hsp110. Of those, plants mainly contain Hsp20, Hsp70 and Hsp90 subfamilies. The genome of *D. catenatum* contains 20 members of Hsp70, whereas there are only 9 in that of *P. equestris.* Interestingly, in particular Hsp70 genes encoding proteins localizing in the cytoplasm have more members in *D. catenatum* than in *P. equestris* (11 vs. 3) (Supplementary Fig. 10). Due to the fact that *D. catenatum* is found in subtropical and temperate regions (Supplementary Fig. 11), can grow in wet and dry environments^4^,^27^, can tolerate both low and high temperatures^4^,^27^, and has a much wider distribution than *P. equestris* (Supplementary Fig. 11), it is interesting to speculate that the additional Hsp70 genes in the *D. catenatum* genome might have helped in the adaptation to a much wider variety of environments. However, more future work will be necessary to prove or disprove this hypothesis.

### Evolution of polysaccharide synthase gene families

**Figure 3:**
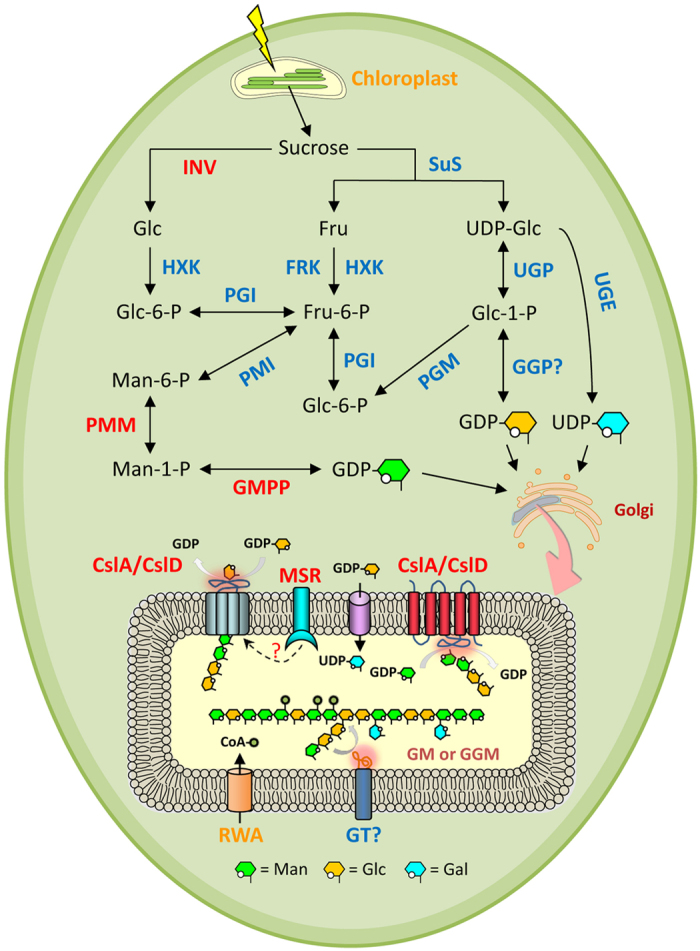
Proposed biosynthetic pathway of GM and GGM in *Dendrobium* stem. The biosynthetic pathway was modified according to the pathways proposed in *Amorphophallus konjac*^31^,^32^. GM or GGM biosynthesis is supposed to be generated from sucrose, mainly produced by photosynthesis in the leaf tissue (Supplementary Note 7). The enzymes indicated in red are highly expressed in the stem. Only abbreviations of gene names are shown: *Csl*, Cellulose synthase like gene; FRK, fructokinase; Fru, fructose; Fru-6-P, Fructose-6-phosphate; GGP, GDP-glucose-pyrophosphorylase; GMPP, GDP-mannose pyrophosphorylase; GT, glycosyltransferase; HXK, hexokinase; INV, invertase; MSR, mannan synthesis-related; PGI, phosphoglucose isomerase; PGM, phosphoglucomutase; PMI, phosphomannose isomerase; PMM, phosphomannomutase; RWA, Reduced Wall Acetylation proten; SuS, sucrose synthase; UGE, UDP-galactose epimerase; UGP, UDP-glucose pyrophosphorylase.

The fleshy stem of *D. catenatum* contains various types of polysaccharides, many of which have medicinal, such as anti-inflammatory, immuno-enhancing, antioxidant and anti-glycation activities^4^,^5^,^28^,^29^. Among those, particularly glucomannan (GM) and galactoglucomannan (GGM) are two major medicinal polysaccharides in *D. catenatum*^30^. Genes involved in GM and GGM biosynthesis were identified through their homology with genes in the Arabidopsis genome. A biosynthetic pathway was proposed and the tissue-specific expression patterns of GM and GGM biosynthesis genes were examined^31^,^32^ (Fig. 3 and Supplementary Note 7). The result suggests that the downstream genes of the biosynthesis pathway are highly expressed in stem tissues where high levels of GM and GGM accumulate. Therefore, we focussed on the analysis of these genes in the *D. catenatum* genome.

Since GM or GGM polysaccharides of *D. catenatum* are easily extracted with water, they may not be tightly bound to the cell wall and probably act as storage polysaccharides in specialized mucilage cells rather than being structural polysaccharides^28^,^30^. Konjac glucomannan (KGM) is water-soluble and accumulates in storage tissues^33^. It also has several bioactivities, such as reducing plasma cholesterol, removing free radicals and inhibiting tumor genesis and metastasis^34^. In addition, the backbone structure of KGM is similar to that of GM. Therefore, we included konjac EST sequences responsible for GM synthesis to search for *D. catenatum* orthologs^32^.

Previous studies showed that *CslA* (*Cellulose synthase-like A*) genes of glycosyltransferase (GT) family 2 are involved in GM backbone synthesis^35^,^36^. We found 13 *CslA* genes in the *D. catenatum* genome, compared to only 6 copies in the *P. equestris* genome. This expansion of *CslA* genes in the *D. catenatum* genome is mainly due to tandem duplication (three arrays: *Dca006365*, *Dca006366*; *Dca007032*, *Dca007033*, *Dca007034*; *Dca013434*, *Dca013437*). Interestingly, these genes were grouped in the same clade with *AkCslA3*, a konjac GM synthase (Supplementary Fig. 12). In addition, two of these genes (*Dca006366* and *Dca007032*) were significantly higher expressed in stem than in other tissues (root, leaf and flower, Supplementary Fig. 13). Therefore, these expanded *CslA* genes may act as GM or GGM synthases in *D. catenatum*. Although *CslD* genes were reported to synthesize mannan rather than GM in *A. thaliana*, a recent study showed that konjac *CslD* may also be involved in the synthesis of GM^31^,^36^. Based on our phylogenetic analysis, two *D. catenatum* genes (*Dca018361* and *Dca000653*) cluster with the konjac *CslD* EST clones (Supplementary Fig. 14). These two genes were highly expressed in stem and leaf, respectively (Supplementary Fig. 15) and suggest their potential roles in the synthesis of GM or GGM.

Arabidopsis *CslD5* was reported to play an important role in osmotic stress tolerance^37^. In addition, GM present in the pseudobulb of an epiphytic CAM orchid, *Cattleya forbesii* Lindl. × *Laelia tenebrosa* Rolfe, has been associated with drought tolerance^38^. The large accumulation of starch, fructan and GM in storage organs of geophytes is critical for their survival in detrimental conditions^39^. Because *Dendrobium* species accumulate high amounts of GM and GGM in their stems and/or leaves, it would be interesting to know whether this is also related to adaptation to environmental stresses, such as drought, a common condition experienced by epiphytic or lithophytic *Dendrobium* species in their natural environment^40^. The online microarray data from Arabidopsis eFP Browser seems to support this because *A. thaliana CslA* (*CslA7* and *CslA10*) and *CslD* (*CslD2* and *CslD3*) genes are induced by drought, osmotic, salt or cold stress (Supplementary Fig. 16).

Storage of carbohydrates in geophytes can serve as carbon and energy sources for the maintenance under adverse environments and for growth under favorable conditions^39^. In addition, soluble sugars, such as glucose and sucrose, can act as osmolytes under osmotic stress^41^. Accumulation of these metabolites is enhanced in response to environmental stresses and has been shown to contribute to drought and freezing tolerance^41^,^42^. In Easter lily bulbs, when stored at –1.0 °C, large amounts of sucrose, mannose, fructose and oligosaccharides accumulated, suggesting that not only starch but also GM was degraded to soluble sugars during frozen storage^43^. Therefore, degradation of GM and GGM to monomers in *Dendrobium* stems might also be induced by stress and play a role in increasing tolerance for drought, cold, salt, and osmosis. GM or GGM were reported to be hydrolysed by glycosyl hydrolase families 5 (GH5) enzymes^44^,^45^,^46^. We thus performed a phylogenetic analysis of *GH5* genes and analysed their tissue-specific gene expression. As the data show, several *DcaGH5* genes were expressed at higher levels in the stems (Supplementary Fig. 17). Among these genes, *Dca014977* clusters with *LeMAN4*, *HvMAN1* and *AtMAN1*, which have been demonstrated to possess hydrolytic activities to GM and GGM^44^,^45^,^46^ (Supplementary Fig. 18). Interestingly, the expression of *AtMAN1* and *OsMAN4*, the rice GH5 gene that grouped with *HvMAN1*, was significantly induced by cold, osmotic, salt or drought stress (Supplementary Fig. 19). All together, these results strongly suggest that the biological functions of GM or GGM in storage organs of *D. catenatum* are related to environmental stress tolerance.

A genome-wide analysis of 12 previously sequenced plant genomes, and subsequent KEGG enrichment analysis of the 629 *D. catenatum* specific gene families (Supplementary Figs 20 and 21; Supplementary Table 15) showed that the functional pathways of these unique families were significantly enriched in ‘Tyrosine metabolism’, ‘Fatty acid metabolism’ and ‘Glycolysis/Gluconeogenesis’ (Supplementary Table 16). Intriguingly, the *D. catenatum* specific genes implicated in the ‘Glycolysis/Gluconeogenesis’ pathway could help to shape and maintain the physiological mechanism that synthesises and stores polysaccharides in the stem.

### Evolution of MADS-box genes

Given that orchids are a unique model system for flower development^12^, we characterised their MADS-box genes, which hold diverse functions in many important processes during plant development, in greater detail. An investigation of the *D. catenatum* genome revealed 63 putative functional MADS-box genes and 12 pseudogenes (Table 1). As earlier reported for *P. equestris*, there seem to be fewer MADS box genes present in orchids than in most other angiosperms, such as rice (*Oryza sativa;* 75 genes) and *A. thaliana* (108 genes). *D. catenatum* has 35 type II MADS-box genes (Table 1), compared with 29 in *P. equestris*. Phylogenetic analysis (Supplementary Fig. 22) shows that most type II MADS-box genes have been duplicated in *D. catenatum*, except for those in the B-*PI* clade. Among these clades, *ANR1* (with three members), *StMADS11* (three members), MIKC* (three members), and *Bs* (two members) contain more members than in *P. equestris* (two members in *ANR1* and one member in other three clades, respectively) (Supplementary Fig. 23). The *ANR1* MADS-box gene in Arabidopsis is a key gene involved in regulating lateral development in response to external nitrate supply^47^. Genes in the *StMADS11* clade have functions in controlling flowering time and inflorescence architecture^48^,^49^. Genes in the *Bs* clade can regulate seed development and fruit size^50^. Recent evidence indicated that the closely related MIKC* MADS-domain proteins are important for the functioning of the *A. thaliana* male gametophyte^51^. However, genes corresponding to the *FLC*, *AGL12* and *AGL15* clades could not be found in the *D. catenatum* genome nor in the *P. equestris* genome. *FLC* genes have recently been found in cereals, although they have proved difficult to identify because they diverged extensively within a relatively short period^52^. However, *AGL12* clade genes are present in the genomes of rice and *A. thaliana,* while *AGL15* clade genes are only present in *A. thaliana*. Therefore, we hypothesise that orthologues of *FLC*, *AGL12* and *AGL15* might have been lost in orchids.

Only 28 putative functional MADS-box type I genes and one pseudogene were found in *D. catenatum* (Supplementary Table 17), suggesting that the *D. catenatum* type I MADS-box genes have experienced a lower birth rate compared with those of type II MADS-box genes. Tandem gene duplication events seem to have contributed to the increase in type I Mα MADS-box genes (Supplementary Fig. 23), indicating that these genes have mainly been duplicated by recent, small-scale duplications^53^. We found that type I Mα MADS-box genes *DcMADS30* and *DcMADS31*, and *DcMADS57* and *DcMADS58* are located side by side in scaffold12110 and scaffold5677, respectively. In addition, three type I Mα MADS-box genes *DcMADS47*, *DcMADS48*, and *DcMADS50* were also found in the same scaffold7526. Interestingly, the *D. catenatum* genome does not contain any type I Mβ MADS-box genes, although these genes do exist in Arabidopsis, poplar and rice. Interactions among type I MADS-box genes are important for the initiation of endosperm development^54^. The failed development of endosperm in orchids might be related to the smaller number of type I MADS box genes in the *D. catenatum* genome.

In conclusion, *Dendrobium* represents a fascinating groups of orchids because of their fleshy stem, various flower architectures, and synthesis of many kinds of different polysaccharides and the *D. catenatum* genome sequence forms an important resource for further exploring orchid gene and genome evolution.

## Method

### Sample preparation and sequencing

For genome sequencing, we collected leaves, stems and flowers from an individual of wild *D. catenatum* (voucher specimen : CHINA.Yunnan: Guangnan county, on rock in evergreen broad-leaf forest, alt. 1350 m,10 March, 2010, Z.J. Liu 4979, NOCC) and extracted genomic DNA using a modified CTAB protocol. Sequencing libraries with insert sizes ranging from 180 bp to 20 Kb (Supplementary Table 1) were constructed using a library construction kit (Illumina, San Diego, CA). These libraries were then sequenced using Illumina HiSeq 2000 platform. The raw reads generated were filtered according to the sequencing quality, presence of adaptor contamination and duplication. Thus, only high-quality reads were used for genome assembly.

### Genome size estimation

To estimate the genome size of *D. catenatum,* we used reads from pair-end libraries to determine the distribution of *K*-mer values. According to the Lander–Waterman theory^55^, the genome size can be determined by the total number of *K*-mers that were divided by the peak value of the *K*-mer distribution. Given the high heterozygosity in the *D. catenatum* genome, we found two peaks in the distribution (Supplementary Fig. 2). Using the second peak as the expected *K*-mer depth, and the formula Genome size = Total *K*-mer/Expected *K*-mer depth, the size of the haploid genome was estimated to be 1.11 Gb (haploid).

### Sequence assembly

Initially, we used SOAPdenovo2^8^ to assemble the genome, which produced an assembly of 1.27 Gb with an N50 scaffold size of 80.56 Kb and a corresponding N50 contig size of 6.64 Kb (Supplementary Table 18). These figures suggest high fragmentation and redundancy. Therefore, to generate a better assembly for further analyses, Platanus^9^, which can effectively manage high-throughput data from heterozygous samples, was used for whole genome shotgun assembly. We subsequently used GapCloser (http://soap.genomics.cn) to fill gaps remaining after the Platanus built-in gap-filling module had been applied. The final assembly was 1.01 Gb in length, approximately 91% of the estimated genome size, with an N50 scaffold size of 391 Kb and a corresponding N50 contig size of 33.1 Kb (Supplementary Table 2).

### Gene and non-coding RNA gene prediction

MAKER^56^ was used to generate a consensus gene set based on *de novo* prediction, homology annotation with CEGMA^10^ and other sequenced monocots, and RNA-seq gene prediction. These results were integrated into a final set of 28,910 protein-coding genes for annotation (Supplementary Table 19). We then generated functional assignments of the *D. catenatum* genes by aligning their CDS (protein-coding sequences) to sequences available in the public protein databases including KEGG^15^, SwissProt^57^, TrEMBL^57^ and InterProScan^58^ (Supplementary Table 20). tRNA genes were searched for by tRNAscan-SE^59^. For rRNA identification, we downloaded the Arabidopsis rRNA sequences from NCBI and aligned them against the *D. catenatum* genome to identify possible rRNAs. Additionally, other types of non-coding RNAs, including miRNA and snRNA, were identified by utilizing INFERNAL^60^ to search from the Rfam database.

### Single nucleotide polymorphisms

We used the BWA program^14^ to remap the pair-end (500 bp) clean reads to the assembled scaffolds. After merging the BAM results, sorting the alignments by the leftmost coordinates and removing potential PCR duplicates, we used SAMtools^15^ ‘mpileup’ to identify single nucleotide polymorphisms (SNPs) and short INDELs. We rejected SNPs and InDels within reads with depths lower (<5 folds) or higher (>80 folds) than expected. Filtering was achieved using the vcfutils.pl varFilter tool in the SAMtools package, with parameters -*Q* 10 -*d* 5 -*D* 86. We estimated heterozygosity rates as the density of heterozygous SNPs from the whole genome, gene intervals, introns and exons.

### Gene family identification

We downloaded genome and annotation data from *Amborella* (*Am.*) *trichopoda* (http://amborella.huck.psu.edu, version 1.0), *Arabidopsis* (*A.*) *thaliana* (TAIR 10), *Brachypodium distachyon* (purple false brome; Phytozome v9.0), *Musaceae acuminata* (http://ensemblgenomes.org, release-21), *Oryza sativa* (Nipponbare, IRGSP-1.0), *Phoenix* (*Ph.*) *dactylifera* (http://qatar-weill.cornell.edu/research/datepalmGenome), *Phalaenopsis equestris* (ftp://ftp.genomics.org.cn/from_BGISZ/20130120/), *Populus* (*Po.*) *trichocarpa* (http://ensemblgenomes.org, release-21), *Sorghum* (*S.*) *bicolor* (sorghum; Phytozome v9.0), *Spirodela* (*Sp.*) *polyrhiza* (common duckweed; http://www.spirodelagenome.org) and *Vitis vinifera* (Phytozome v9.0), *Zea mays* (http://www.plantgdb.org/ZmGDB), *Phyllostachys* (*Phy.*) *heterocycla* (http://www.bamboogdb.org). We chose the longest transcript to represent each gene, and removed gene models with an open reading frame (ORF) shorter than 150 bp. These protein sets were aligned and clustered using OrthoMCL^61^.

### Phylogenomic dating

We conducted phylogenomic dating with PAML McMcTree^62^. The McMc process was run for 1,500,000 iterations, with a sample frequency of 150 after a burn-in of 500,000 iterations. Other parameters used the default settings of McMcTree. Two independent runs were performed to check convergence. The following constraints were used for time calibrations:

1. 140–150 million years ago (MYA) for the monocot – dicot split^63^,
2. 94 MYA as the lower boundary for the *Vitis* – Eurosid split^52^,
3. 130 MYA as the lower boundary for the Alismatales – Acorales and core monocots (Commelinids, Asparagales, Liliales, etc.) split^64^, and
4. 200 MYA as the upper boundary for basal angiosperms^65^.

Based on these divergence time ranges and the inferred phylogenetic tree, the divergence times between the 12 species were estimated using McMcTree software.

### Identification of resistance genes

HMMER V3.0 was used to align the protein sequences of *D. catenatum* against the hidden Markov model of the Pfam NBS (NB-ARC). The TIR and LRR domains were detected by using the Pfam_Scan (−E 0.01 –domE 0.01). MARCOIL^66^ and paircoil2^67^ were utilized for identification of the CC motif.

### Identification of polysaccharide-related genes

We collected polysaccharide-related genes of Arabidopsis first by using the CAZY database and other information resources. Then, we performed TBLASTN search against all coding sequences (CDS) datasets of each plant species. These CDS datasets were downloaded from Phytozome (poplar, Selaginella and Physcomitrella), ConGenIE (Norway spruce), QATAR-WEILL.CORNELL (dates palm), RAP-DB (rice) and TAIR (Arabidopsis). In case of *Amo. Konjac*, RNA-seq data in NCBI SRA (accession number SRX098311) was downloaded and assembled by CLC genomic workbench software. Homologous genes from these species with BLAST E-values less than 1e-5 were then used as BLASTX queries against all protein sequences in Arabidopsis. If the top-hit genes of this BLASTX results belonged to polysaccharide-related genes defined previously, the queries were defined as orthologues in each species. The phylogenetic trees of collected orthologs were constructed by ClustalW^68^.

### Evolution of MADS box genes in *D. catenatum*

The MADS-box domain is comprised of 60 amino acids, which we identified for all the potential MADS-box sequences of *D. catenatum*. Next, we aligned all the MADS-box genes with ClustalW. An un-rooted neighbour-joining phylogenetic tree was constructed in MEGA5^69^ with default parameters. Confidence on the tree branches was evaluated by bootstrap analysis (1000 replicates).

Associated references and supplementary information are available in the online version of the paper.

## Additional Information

**Accession codes**: Genome sequences have been submitted to the National Center for Biotechnology Information (NCBI). Whole genome assemblies have been deposited in DDBJ/EMBL/GenBank under the accession codes JSDN00000000 (URL: http://www.ncbi.nlm.nih.gov/bioproject/262478).

**How to cite this article**: Zhang, G.-Q. *et al.* The *Dendrobium catenatum* Lindl. genome sequence provides insights into polysaccharide synthase, floral development and adaptive evolution. *Sci. Rep.* **6**, 19029; doi: 10.1038/srep19029 (2016).

## Supplementary Material

Supplementary Information

Supplementary Table 12

Supplementary Table 13

## Figures and Tables

**Table 1:**
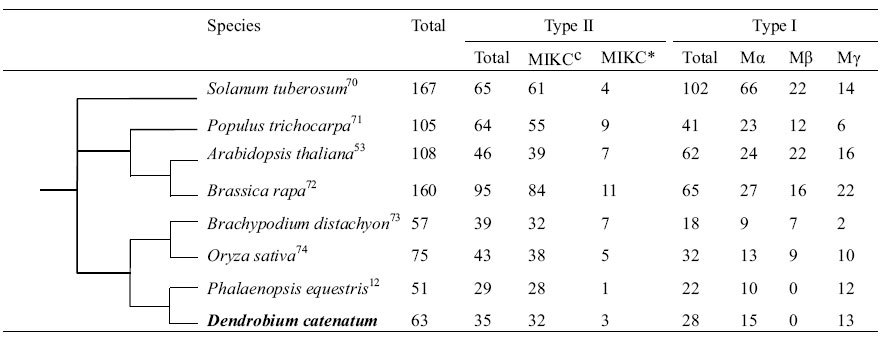
The number of MADS-box genes in some representative plant species.
