# Supplementary material for: The Dendrobium catenatum Lindl. genome sequence provides insights into polysaccharide synthase, floral development and adaptive evolution
Source: Sci Rep. 2016 Jan 12;6:19029. doi: 10.1038/srep19029 (PMC4709516; doi:10.1038/srep19029)
Supplement: Supplementary Information [file srep19029-s1.pdf]

Supplementary Information

The *Dendrobium catenatum* Lindl. genome sequence provides insights into polysaccharide synthase, floral development and adaptive evolution

Guo-Qiang Zhang[1*], Qing Xu[2*], Chao Bian[3*], Wen-Chieh Tsai[4, 5, 6*], Chuan-Ming Yeh[7*], Ke-Wei Liu[8*], Kouki Yoshida[9*], Liang-Sheng Zhang[10*], Song-Bin Chang[4], Fei Chen[11], Yu Shi[1, 12], Yong-Yu Su[1, 12], Yong-Qiang Zhang[1], Li-Jun Chen[1], Yayi Yin[1], Min Lin[1], Huixia Huang[1], Hua Deng[13], Zhi-Wen Wang[14], Shi-Lin Zhu[14], Xiang Zhao[14], Cao Deng[14], Shan-Ce Niu[2], Jie Huang[1], Meina Wang[1], Guo-Hui Liu[1], Hai-Jun Yang[1, 12], Xin-Ju Xiao[1], Yu-Yun Hsiao[5], Wan-Lin Wu[1,5], You-Yi Chen[4, 5], Nobutaka Mitsuda[15], Masaru Ohme-Takagi[7, 15], Yi-Bo Luo[2†], Yves Van de Peer[16,17,18†], Zhong-Jian Liu[1, 8, 12†]

*These authors contributed equally to this work.

Correspondence and requests for materials should be addressed to Z-J.L. (liuzj@sinicaorchid.org), Y. V. d. P. (yves.vandepeer@psb.vib-ugent.be), Y-B.L. (luoyb@ibcas.ac.cn).

Content

Supplementary Notes … 5

- Supplementary Note 1. Plant material … 5
- Supplementary Note 2. Chromosome preparation … 5
- Supplementary Note 3. Transposable element analysis … 6
- Supplementary Note 4. Gene prediction … 6
- Supplementary Note 5. Gene family annotation … 7
- Supplementary Note 6. Collinearity identification and WGD time estimation … 8
- Supplementary Note 7. Proposed biosynthetic pathway of GM and GGM in *D. catenatum* stem … 8

Supplementary Figures … 10

- Supplementary Figure 1. Propidium iodide (PI) stained chromosomes at metaphase, prometaphase and interphase stages. … 10
- Supplementary Figure 2. Estimation of genome size based on 17-mer distribution … 11
- Supplementary Figure 3. Comparison of the lengths of scaffolds assembled using the SOAPdenovo2 and Platanus software programs, respectively … 12
- Supplementary Figure 4. Distribution of the sequencing depth of the assembled genome of *D. catenatum*. … 13
- Supplementary Figure 5. Distribution of divergence times for the complete long terminal repeats (LTRs) in *D. catenatum*. … 14
- Supplementary Figure 6. Gene models were supported by evidence from *de novo* prediction, protein-based homology searches and RNA-seq data. … 15
- Supplementary Figure 7. Gene length distribution for seven plant species. … 16
- Supplementary Figure 8. Gene family expansion and contraction in different flowering plant lineages, mapped on the phylogenetic tree of Figure 1. … 17
- Supplementary Figure 9. Distribution of single nucleotide polymorphisms (SNPs) in the *D. catenatum* genome … 18
- Supplementary Figure 10. Evolution of selected heat shock protein (*Hsp*) gene families. … 19
- Supplementary Figure 11. Comparison of distributions of *D. catenatum* and *P. equestris* (modified from Cribb[19] and drawn by Li-Jun Chen using Photoshop 8.0.1[20]) … 20
- Supplementary Figure 12. Phylogenetic tree of *CslA*. … 21
- Supplementary Figure 13. Heat map showing the expression of *CslA* genes in four tissues of *D. catenatum* … 22
- Supplementary Figure 14. The phylogenetic tree of *CslD*. … 23
- Supplementary Figure 15. Heat map showing the transcriptome expression of *CslD* genes in four tissues of *D. catenatum*. … 24
- Supplementary Figure 16. Expression levels of Arabidopsis *CslA* and *CslD* genes in response to abiotic stresses … 25
- Supplementary Figure 17. Heat map showing the transcriptome expression of *GH5* genes in four tissues of *D. catenatum* … 26
- Supplementary Figure 18. The phylogenetic tree of *GH5* gene families. … 27
- Supplementary Figure 19. Expression levels of Arabidopsis and rice *GH5* genes in response to abiotic stresses … 28
- Supplementary Figure 20. Orthologous genes found in different plant species. … 29

Supplementary Figure 21. Venn diagram showing unique and shared gene families among members of Orchidaceae, dicots and Poaceae, and *M. acuminata* and *Ph. dactylifera* 30
Supplementary Figure 22. Phylogenetic tree of Type II MADS-box genes from *O. sativa* (Os), *A. thaliana* (At), *P. equestris* (PEQU) and *D. catenatum* (Dc) 31
Supplementary Figure 23. Phylogenetic tree of Type I MADS-box genes from *O. sativa* (Os), *A. thaliana* (At), *P. equestris* (PEQU) and *D. catenatum* (Dc). 32
Supplementary Figure 24. Phylogenetic tree of *Dendrobium* based on nr ITS and plastid DNA 33
Supplementary Figure 25. Pearson's correlation analysis between intron length and intronic TE length for............34
Supplementary Figure 26. Five-way Venn diagrams showing unique and shared gene families among different plant species............35
Supplementary Tables............36
Supplementary Table 1. Summary of data generated for the *D. catenatum* genome sequencing with HiSeq 2000[a] 36
Supplementary Table 2. Summary of the *D. catenatum* genome assembly with Platanus............37
Supplementary Table 3. CEGMA evaluation for the completeness of the *D. catenatum* genome assembly............38
Supplementary Table 4. Evaluation of the *D. catenatum* genome completeness using data set of RNA transcripts............39
Supplementary Table 5. Statistics for repetitive elements in the *D. catenatum* genome............40
Supplementary Table 6. Gene models supported by differing evidence types 41
Supplementary Table 7. Statistics of gene element length for seven sequenced plant species ..42
Supplementary Table 8. Statistics for noncoding RNAs in *D. catenatum*............43
Supplementary Table 9. GO term enrichment results of significantly expanded gene families in the *D. catenatum* lineage............44
Supplementary Table 10. KEGG pathway enrichment results for SNP-related genes............45
Supplementary Table 11. GO term enrichment results for SNP-related genes............46
Supplementary Table 12. Tandem duplicated genes in *D. catenatum* shown as gene pairs............48
Supplementary Table 13. Tandem duplicated genes in *P. equestris* shown as gene pairs 48
Supplementary Table 14. List of the resistant genes of *D. catenatum* and *P. equestris* 49
Supplementary Table 15. Summary of orthologous gene families in 12 sequenced plant species 50
Supplementary Table 16. List of the putative genes involved in GM/GGM synthesis and hydrolysis in the *D. catenatum* genome............51
Supplementary Table 17. List of the 75 MADS-box genes identified in *D. catenatum*............53
Supplementary Table 18. Summary of the *D. catenatum* genome assembly obtained with SOAPdenovo2 55
Supplementary Table 19. Statistics of annotation results from various prediction methods............56
Supplementary Table 20. Statistics for gene function assignments from different databases 57
Supplementary Table 21. Enriched KEGG pathways for *D. catenatum* specific gene families.58
Supplementary Table 22. GO term enrichment results for Orchidaceae-specific gene families.59
Supplementary Table 23. KEGG pathway enrichment results for Orchidaceae-specific gene families............60

Supplementary Table 24. GO term enrichment results for monocot-specific gene families 61
Supplementary Table 25. KEGG pathway enrichment results for monocot-specific gene families 62
Supplementary Table 26. The sequences of konjac EST clones used in the *CslA* and *CslD* phylogenetic trees 63
Supplementary References 64

Supplementary Notes

Supplementary Note 1. Plant material

Dendrobium catenatum is often confused with similar species such as *D. scoriarum* W. W. Smith, *D. moniliforme* (Linnaeus) Swartz, *D. huoshanense* C. Z. Tang & S. J. Cheng, and with many artificial hybrids that are cultured to produce health food[1, 2]. The closest sister species of *D. catenatum* is considered to be *D. scoriarum* (**Supplementary Figure 24**). Given that hybridisation is common in cultivation, to ensure that a true *D. catenatum* and not a hybrid, such as *D. catenatum* × *D. scoriarum*, was used as material for genome sequencing, we collected plant samples of *D. catenatum* from the wild as permitted by the Chinese Government, in 2010. We collected wild plants from Guangnan in Yunnan, Xinning in Hunan and Langshan in Hunan, China. The characteristics of the collected plants were consistent with those of *D. catenatum* as described in the *Flora of China* 25[1] and *The Dendrobiums*[3], morphologically confirming that our samples were indeed *D. catenatum*. We also conducted molecular biological identification: nuclear and plastid markers were subjected to phylogenetic analysis, which suggested that the samples from all three origins were the same species and were sisters to *D. scoriarum*, confirming at the molecular level that our samples were *D. catenatum* (**Supplementary Figure 24**). The plants from Guangnan were used as material for genome sequencing.

Supplementary Note 2. Chromosome preparation

The newly growing root tips were excised and treated with 2 mM 8-hydroxyquinolino for 3 h at 15 ℃. The root tips were then fixed in freshly prepared Carnoy’s solution (3:1 (v/v) 95 % ethanol/glacial acetic acid) and stored at -20 ℃. The fixed material was washed with distilled water and digested with an enzyme mixture containing 1% pectinase solution (Sigma), 1% pectolyase Y23 and 1% Cellulase RS (Yakult) in 10mM citrate buffer (40 mM citric acid, 60 mM tri-sodium citrate, pH 4.5) for 30–40 min. Next, we carefully rinsed fragile meristem with distilled water, squashed in a drop of 45 % acetic acid and removed the coverslip after freezing in

liquid nitrogen. The slides were dried at 42°C and rinsed with Carnoy's solution and 95 % ethanol. Finally, chromosomes were stained with 10 µg/ml propidium iodide (PI) of 15 µl VECTASHIELD® Mounting Media (Vector Laboratories, Burlingame, United States) and chromosome morphology could be observed under the fluorescence microscope (Nikon eclipse 80i). The images of chromosome complements or interphase cells were captured with a CCD camera (Nikon DS Ri1) and then analyzed with Nikon NIS-Elements software (**Supplementary Figure 1**).

Supplementary Note 3. Transposable element analysis

The percentage of genes lacking transposable elements (TEs) within introns was 13.69% for *D. catenatum*, which is lower than for banana (*Musa acuminata*; 65.45%), date palm (*Phoenix dactylifera*; 58.74%), rice (*Oryza sativa*; 52.21%) and *Arabidopsis thaliana* (90.10%). Given that this large proportion of TEs occurs in combination with an extraordinary long average intron length, we hypothesised a correlation between these parameters. A detailed distribution of the TE rate in introns (**Supplementary Figure 5**) also shows an extra peak of approximately 50% in the TE ratio in the introns of *D. catenatum*, which is not present in other species. To explore how the accumulation of TEs affects the length of introns, we conducted a Pearson's correlation analysis on four plant species (**Supplementary Figure 25**). We found that the longer the average intron, the stronger the correlation between intron length and intronic TE length, culminating in an almost complete correlation in *D. catenatum*. Therefore, we conclude that the increased intron length observed in *D. catenatum* is mainly the result of intronic TE insertion. We observed a higher-than-average percentage of transposable elements (TEs) in the genome of *D. catenatum* compared to other plant species. This was shown (**Supplementary Note 4 and Supplementary Table 7**) to cause the overall great intron length in the *D. catenatum* genome.

Supplementary Note 4. Gene prediction

MAKER[5] was used to generate a consensus gene set based on *de novo* prediction,

homology annotation with CEGMA[6] and other sequenced monocots, and RNA-seq gene prediction. These results were integrated into a final set of 28,910 protein-coding genes for annotation **(Supplementary Table 19)**. *D. catenatum* was found to have a longer average gene length than most other sequenced plants, but similar to that of *P. equestris* **(Supplementary Figure 7 and Table 7),** because both species have a long average intron length. Therefore, this feature might be a unique characteristic of Orchidaceae. We were able to generate functional assignments for 83.15% of the *D. catenatum* genes from at least one of the public protein databases **(Supplementary Table 19)**.

Supplementary Note 5. Gene family annotation

Gene family clustering was performed based on the set of 28,910 predicted genes from *D. catenatum* with the protein-coding genes of seven other monocots (*P. equestris, S. bicolor, B. distachyon, O. sativa, M. acuminata* and *Ph. dactylifera*), three dicots (*Po. trichocarpa, A. thaliana* and *V. vinifera*) and the outgroup *Am. trichopoda*. This analysis yielded 13,530 gene families in *D. catenatum*, containing 21,857 predicted genes (75.6% of the total genes identified; **Supplementary Figure 20 and Supplementary Table 15)**. A four-way comparison of Orchidaceae, dicots, Poaceae, *M. acuminata* and *Ph. dactylifera* (**Supplementary Figure 21**) uncovered 10,052 gene families to be shared by all species, with 2,859 gene families unique to Orchidaceae, which is fewer than for the dicots (3,884) or Poaceae (5,668). Two five-way comparisons were also performed to show the number of species-specific gene families, and also uncovered more gene families in Poaceae (**Supplementary Figure 26**).

For the *D. catenatum*-specific gene families, we conducted a GO/KEGG enrichment analysis and found enrichment of the KEGG pathways ‘Tyrosine metabolism’, ‘Fatty acid metabolism’ and ‘Glycolysis/Gluconeogenesis’ (**Supplementary Table 21**). On further analysis of the Orchidaceae- and monocot-specific gene families, Orchidaceae-specific gene families were found to be enriched

for GO terms associated with transcription (**Supplementary Table 22**) and the pathways 'GPI-anchor biosynthesis' (**Supplementary Table 23**), whereas the monocot-specific gene families were enriched for the GO terms 'ADP binding,' 'defence response', 'solute:hydrogen antiporter activity', 'two-component response regulator activity' and the KEGG pathways 'RNA polymerase', 'Pyrimidine metabolism', 'Purine metabolism', 'Flavonoid biosynthesis' and 'Stilbenoid, diarylheptanoid and gingerol biosynthesis' (**Supplementary Tables 24** and **25**).

We constructed a phylogenetic tree based on a concatenated sequence alignment of 677 single-copy gene families from *D. catenatum* and 11 other plant species by using PhyML[7] software with Maximum Likelihood method (**Figure 1**).

Supplementary Note 6. Collinearity identification and WGD time estimation

A total of 14,289 *D. catenatum* genes reside on scaffolds of fewer than 20 genes, of which approximately 4,982 genes are located on scaffolds with fewer than five genes; all are of limited use for the intragenome detection of collinearity. Therefore, we believe the collinearity of the genome is likely to constitute a substantial underestimate.

For every paralogue in the *D. catenatum* genome, we filtered out all of the tandem gene pairs and then calculated the K_s value for each pair. A correction was performed using the method described by Maere et al.[9]. Divergence time estimates were based on histogram peak K_s values of the youngest pairs and a molecular clock of λ=6.5 $\times10^{-9}$ synonymous substitutions per site per year[8].

Supplementary Note 7. Proposed biosynthetic pathway of GM and GGM in *D. catenatum* stem

The biosynthetic pathway we proposed for GM and GGM was modified from the one proposed for *Amorphophallus konjac*[10]. GM or GGM biosynthesis is generated from sucrose produced by photosynthesis in the *D. catenatum* leaf and/or stem. The enzymes indicated in red are highly expressed in the stems (**Figure 3**,

Supplementary Figure 16 and 19). The topology of *Csl* proteins is unknown. The *CslA* and *CslD* enzymes may contain multiple transmembrane domains (**Supplementary Table 16**) and probably have catalytic sites (indicated in pink) in either cytosolic or the luminal side of Golgi membrane. In case the catalytic site is located in the Golgi apparatus, NDP-sugar transporters are necessary for the transportation of NDP-sugars from the cytoplasm to the Golgi apparatus[11]. There are 19 putative NDP-sugar transporter genes in the *D. catenatum* genome (**Supplementary Table 16**). The Glc and Man in GGM backbone can be modified with galactose (Gal) side chains in α-1,6-linkage[12]. In addition, GGM also contains side branching points at the C6 position of Glc[13,14] or Man[15] by β-1,6-linkage. However, the glycosyltransferase (GT) family genes responsible for this branching formation are still unknown. Furthermore, the O-2 positions of some Man of GGM are modified with acetyl groups[14] and Reduced Wall Acetylation (RWA) gene acts as a probable acetyl-CoA transporter[16]. Three putative RWA genes were found in the *D. catenatum* genome (**Supplementary Table 16**). This acetylation has been proved to be essential for GGM medicinal activity in some *Dendrobium* species[17]. Mannan synthesis-related (MSR) proteins have a single transmembrane domain and have been demonstrated to be localized in the Golgi apparatus. It is involved in GM or mannan synthesis in an unknown manner[12]. A previous study hypothesized that MSR may be involved in the synthesis of primer of GM, which is important for the initiation of GM synthesis such as xylans, a hemicellulosic polysaccharide[16, 18]. One *MSR* candidate gene, highly expressed in stem, was isolated from the *D. catenatum* genome (**Supplementary Table 16**). It would be interesting to know whether this *MSR* gene also plays an important role in the biosynthesis of GM or GGM.

Supplementary Figures

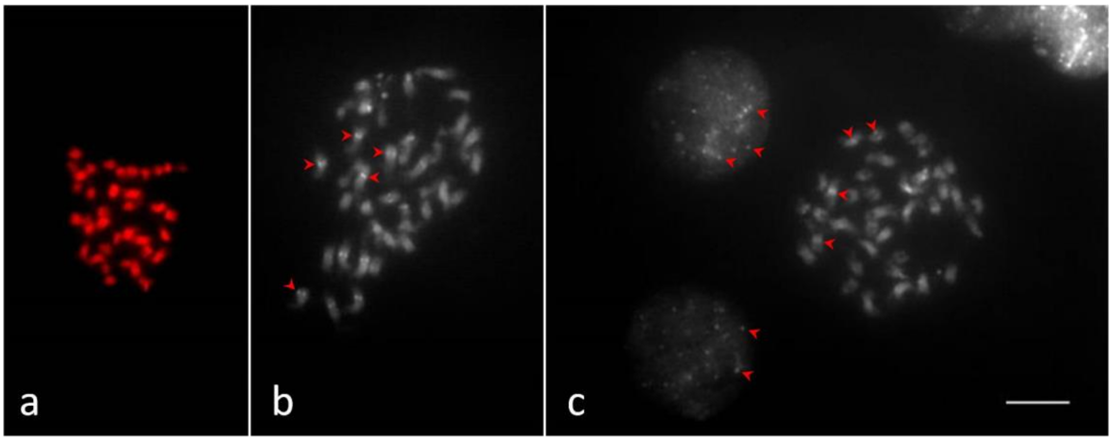

Supplementary Figure 1. Propidium iodide (PI) stained chromosomes at metaphase, prometaphase and interphase stages. A metaphase complement of 2n = 38 chromosomes (red pseudo-colored) with sizes of approximately 2 µm (a). Small dots (indicated only some by arrowheads) consistently showed constitutive heterochromatin at prometaphase chromosomes (b and c) and interphase nuclei (c). Bar represents 10 µm.

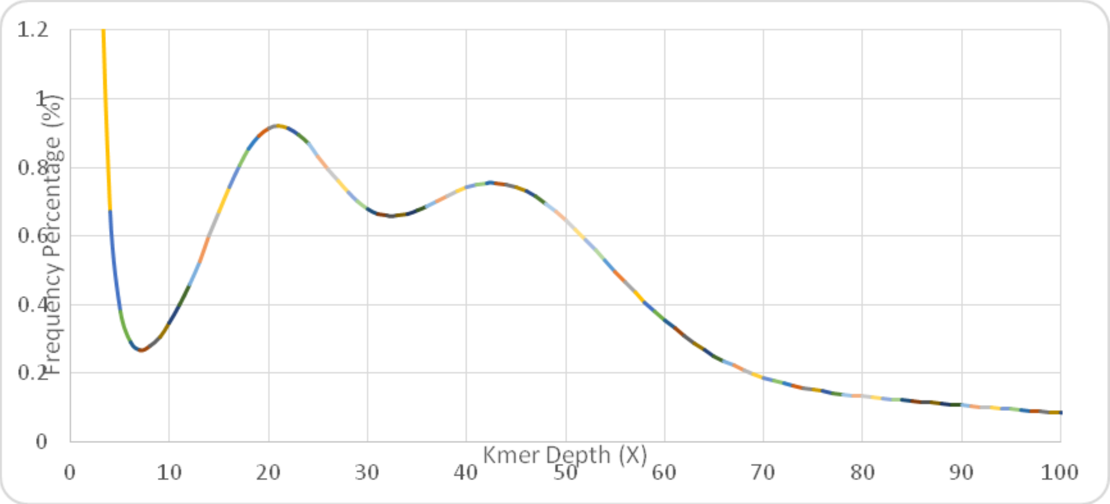

Supplementary Figure 2. Estimation of genome size based on 17-mer distribution. The first peak appearing at 21 is caused by the high amount of heterozygosity. Genome size can be estimated using the position of the second peak: the total number of K-mers is 46,613,336,744, and the position of the second peak is at 42; therefore, the genome size of *D. catenatum* is estimated to be 1,109,841,351 (=46,613,336,744/42).

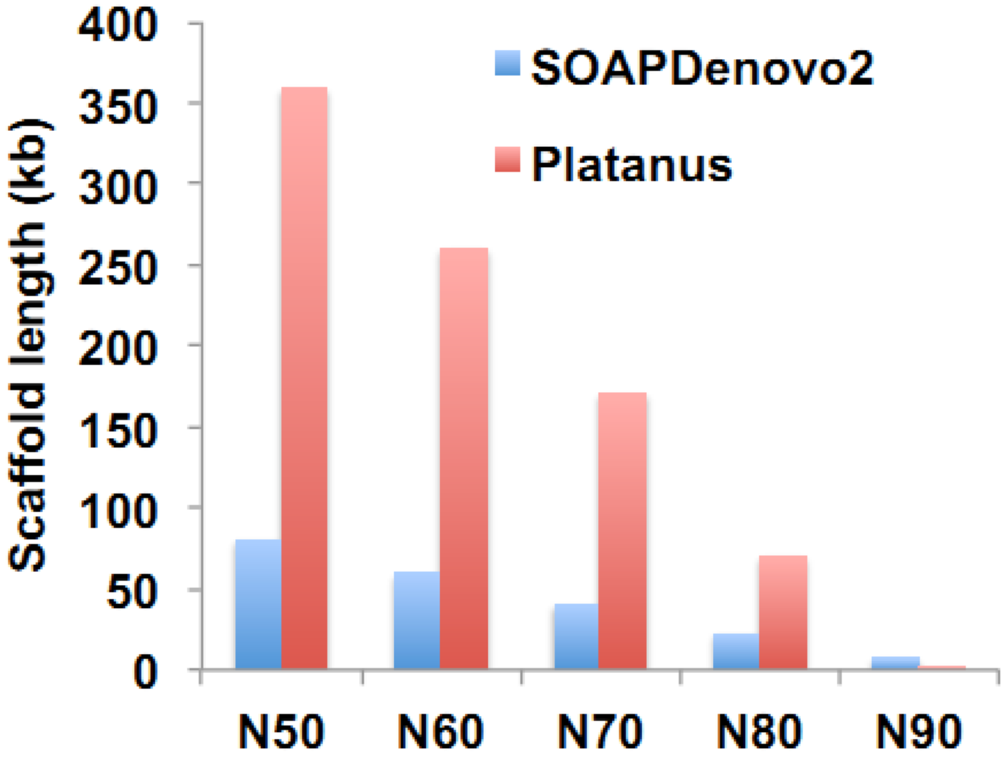

Supplementary Figure 3. Comparison of the lengths of scaffolds assembled using the SOAPdenovo2 and Platanus software programs, respectively

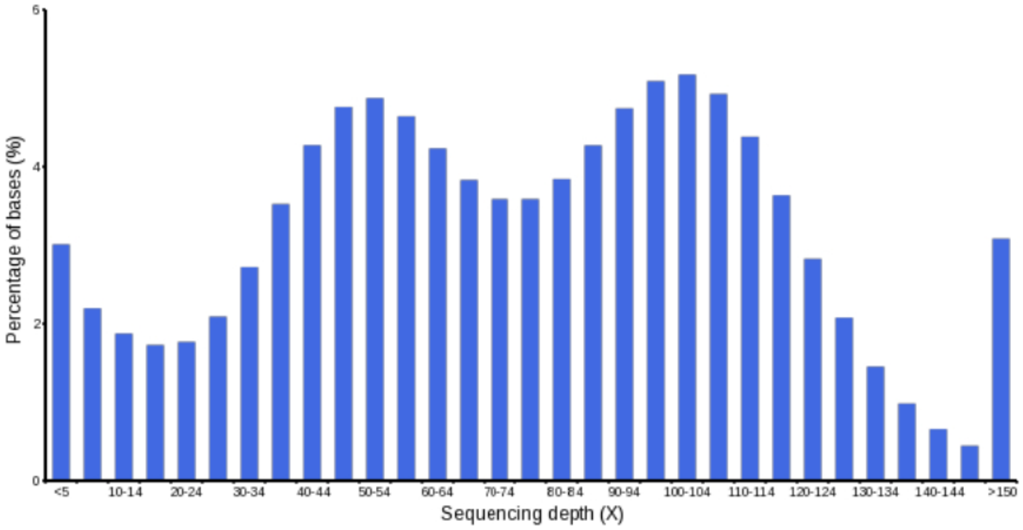

Supplementary Figure 4. Distribution of the sequencing depth of the assembled genome of *D. catenatum*. Mapping all of the paired-end reads to the assembly reveals that 97% of the sequence has a coverage depth greater than five.

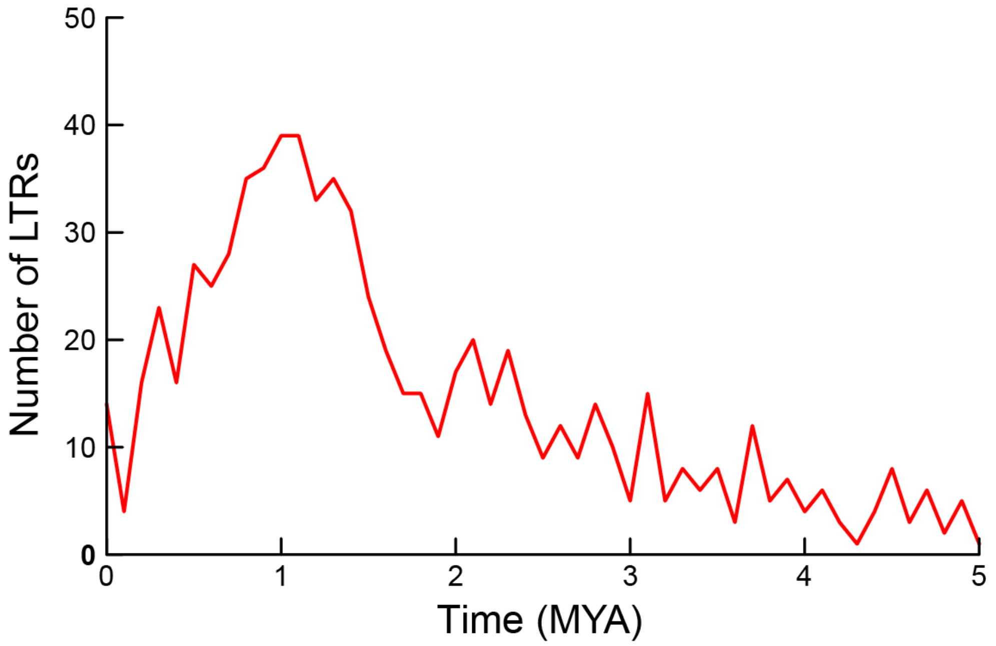

Supplementary Figure 5. Distribution of divergence times for the complete long terminal repeats (LTRs) in *D. catenatum*. The results indicate that a burst of LTR activity occurred during the last five million years. MYA, million years ago.

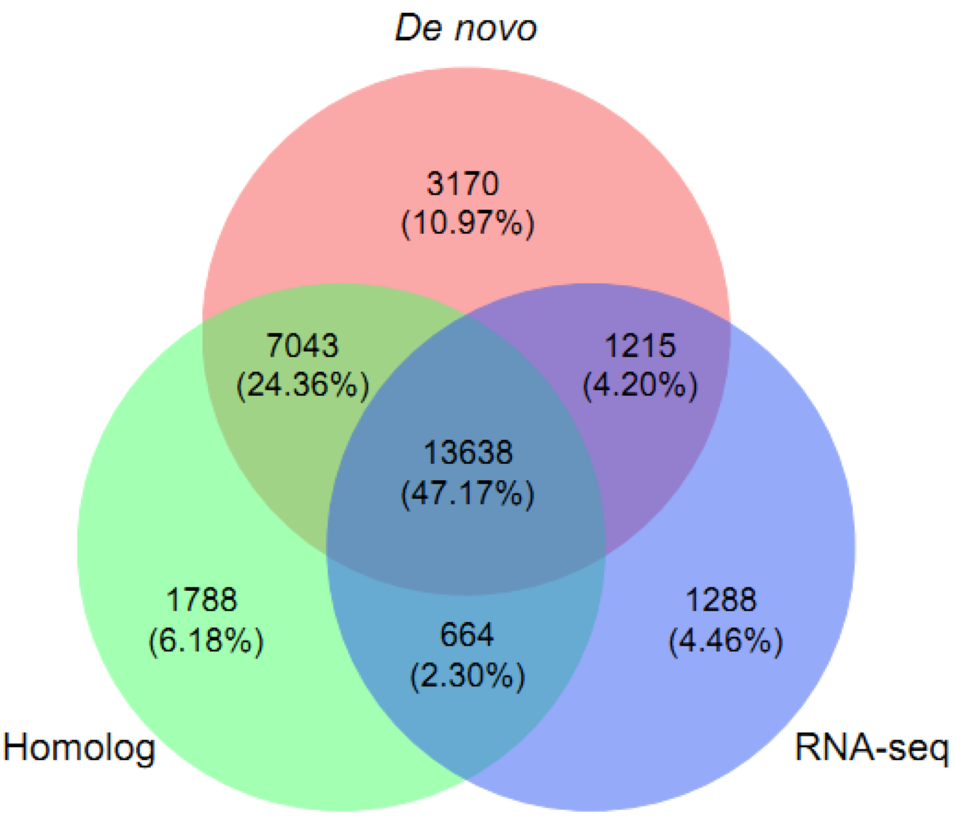

Supplementary Figure 6. Gene models were supported by evidence from *de novo* prediction, protein-based homology searches and RNA-seq data. The results indicate that 22,394 (74.9%) of 28,910 annotated protein-coding genes are supported by transcriptome data.

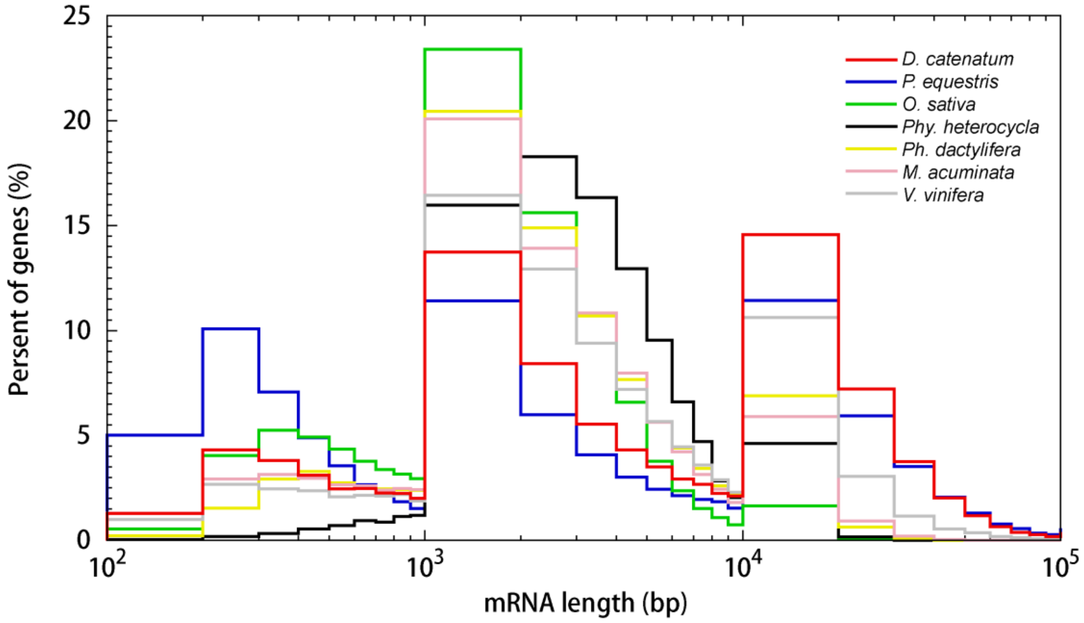

Supplementary Figure 7. Gene length distribution for seven plant species. *D. catenatum* has, on average, longer genes than most other sequenced plant species. See text for details.

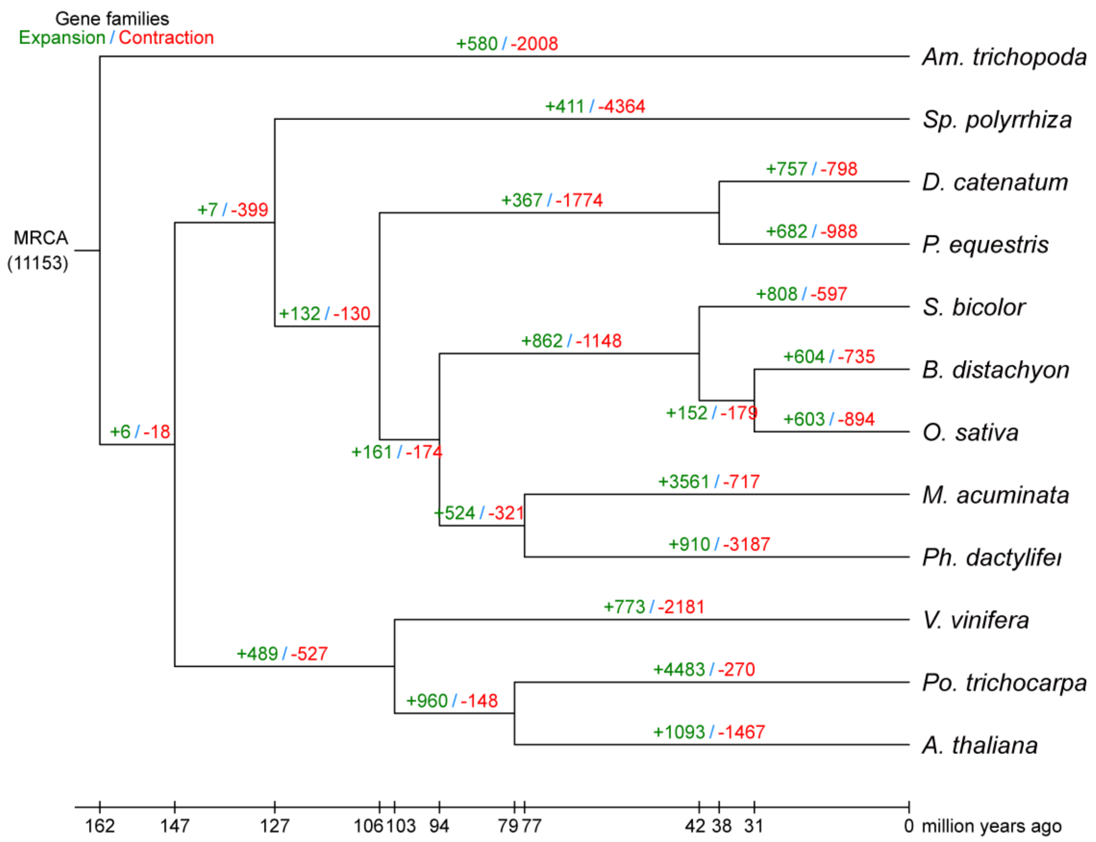

Supplementary Figure 8. Gene family expansion and contraction in different flowering plant lineages, mapped on the phylogenetic tree of Figure 1. Expanded gene families are shown in green, while contracted gene families are shown in red. MRCA, most recent common ancestor.

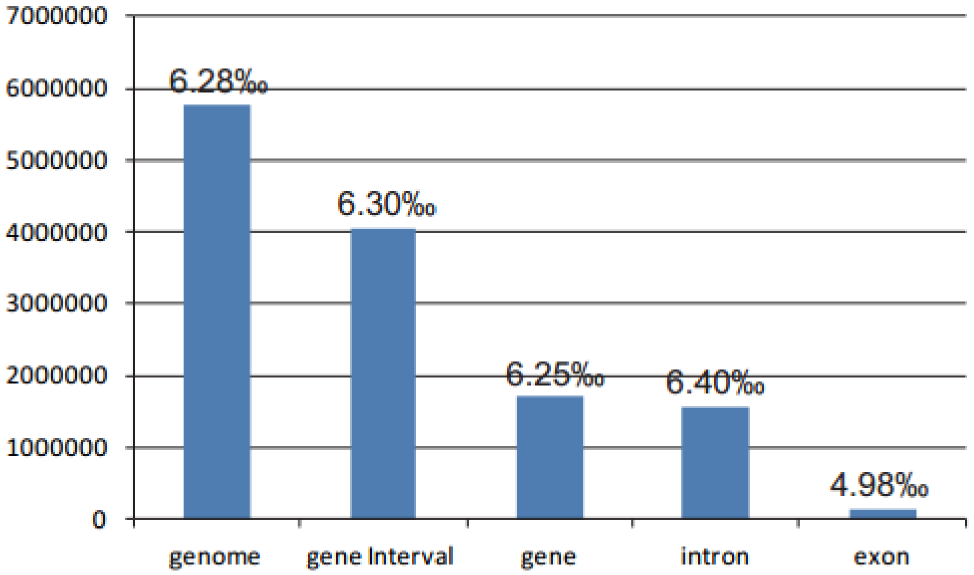

Supplementary Figure 9. Distribution of single nucleotide polymorphisms (SNPs) in the *D. catenatum* genome. The heterozygous SNP rate for the whole genome was estimated at 6.28×10^{-3}, whereas the SNP rate in exons was estimated to be 4.98×10^{-3}. Thousandths at each bar indicate the SNP ratio in each region.

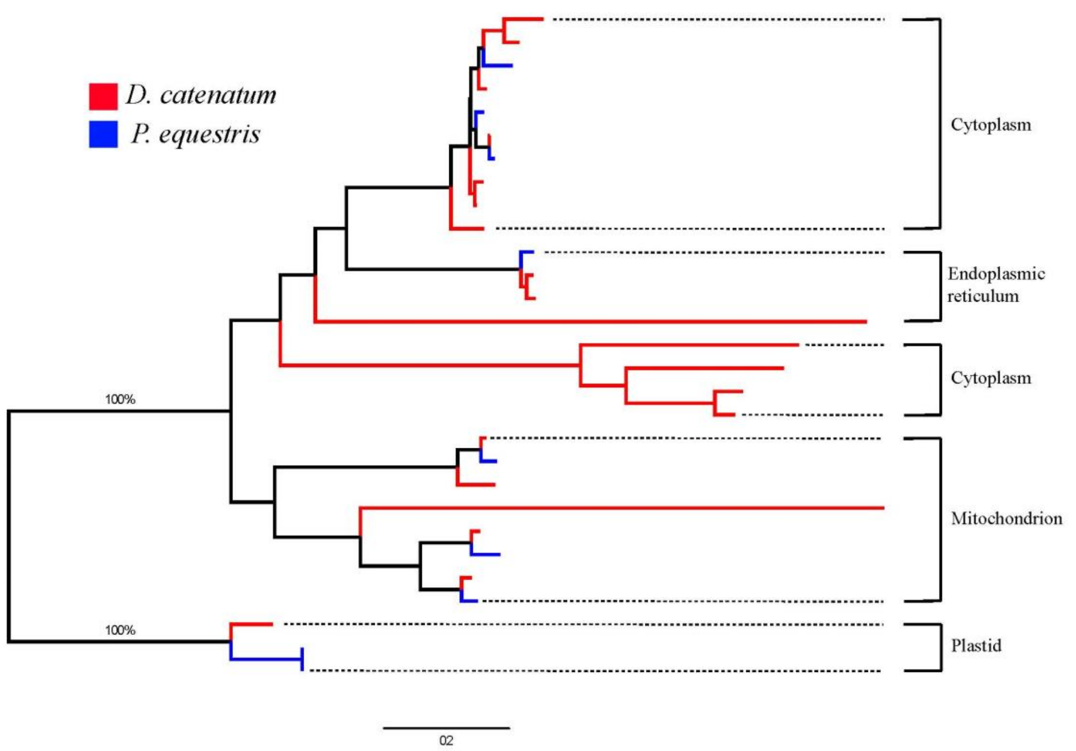

Supplementary Figure 10. Evolution of selected heat shock protein (*Hsp*) gene families. Hsp70 gene products localizing in the cytoplasm have more members in *D. catenatum* than in *P. equestris* (11 vs. 3). Red branches represented Hsp genes of *D. catenatum* while blue branches represent *P. equestris*.

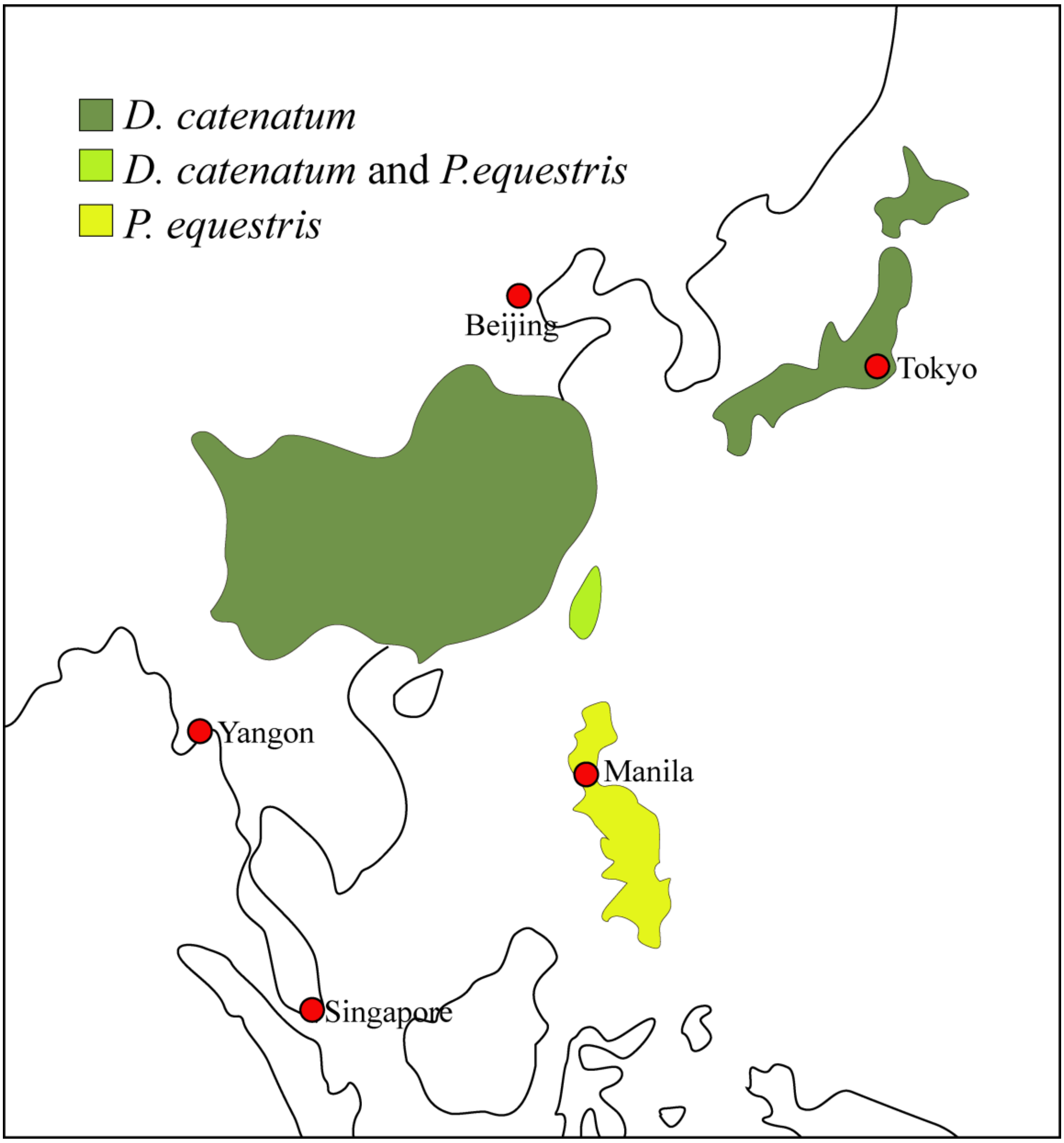

Supplementary Figure 11. Comparison of distributions of *D. catenatum* and *P. equestris* (modified from Cribb[19] and drawn by Li-Jun Chen using Photoshop 8.0.1[20]). *D. catenatum* is found in subtropical and temperate regions and has a much wider distribution than *P. equestris*.

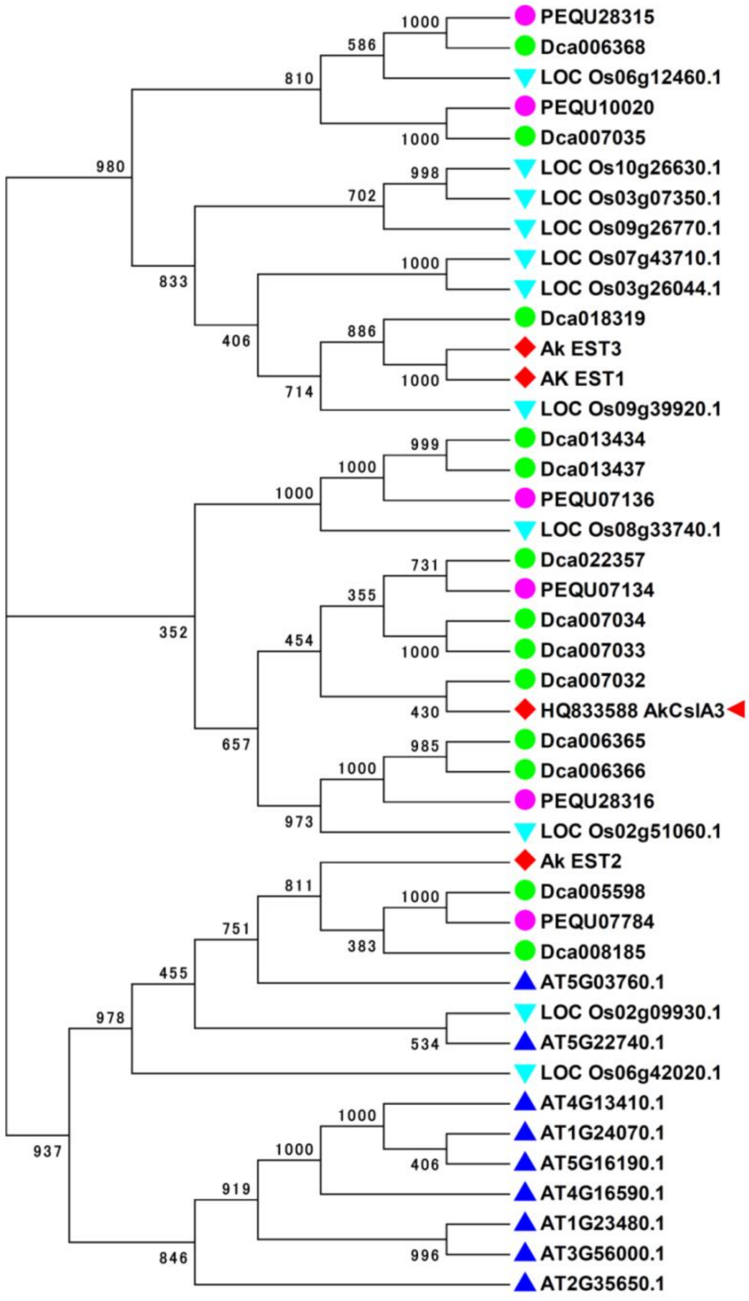

Supplementary Figure 12. Phylogenetic tree of *CslA*. The AkCslA3 gene with proved glucomannan synthase activity is indicated by a red arrowhead. The light and deep blue triangles indicate rice (labeled LOC) and A. thaliana (AT), respectively. The green and pink dots represent *D. catenatum* (Dca) and *P. equestris* (PEQU), respectively. All sequences used here are longer than 200 amino acids. The konjac EST sequences are provided in **Supplementary Table 26**. Bootstrap values are shown on each branch.

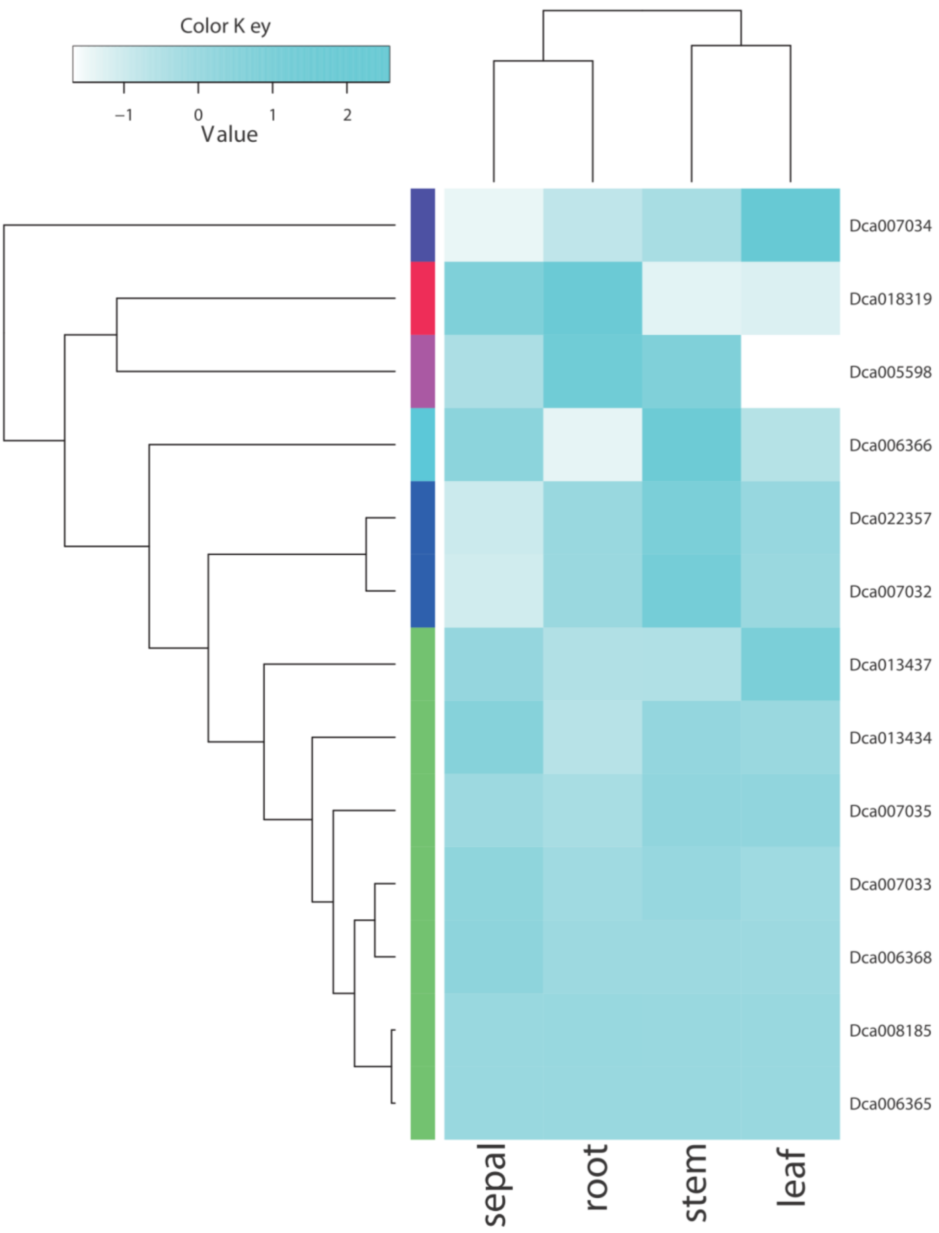

Supplementary Figure 13. Heat map showing the expression of *CslA* genes in four tissues of *D. catenatum*. Genes shown in dark cyan are highly expressed, genes shown in light cyan are lowly expressed.

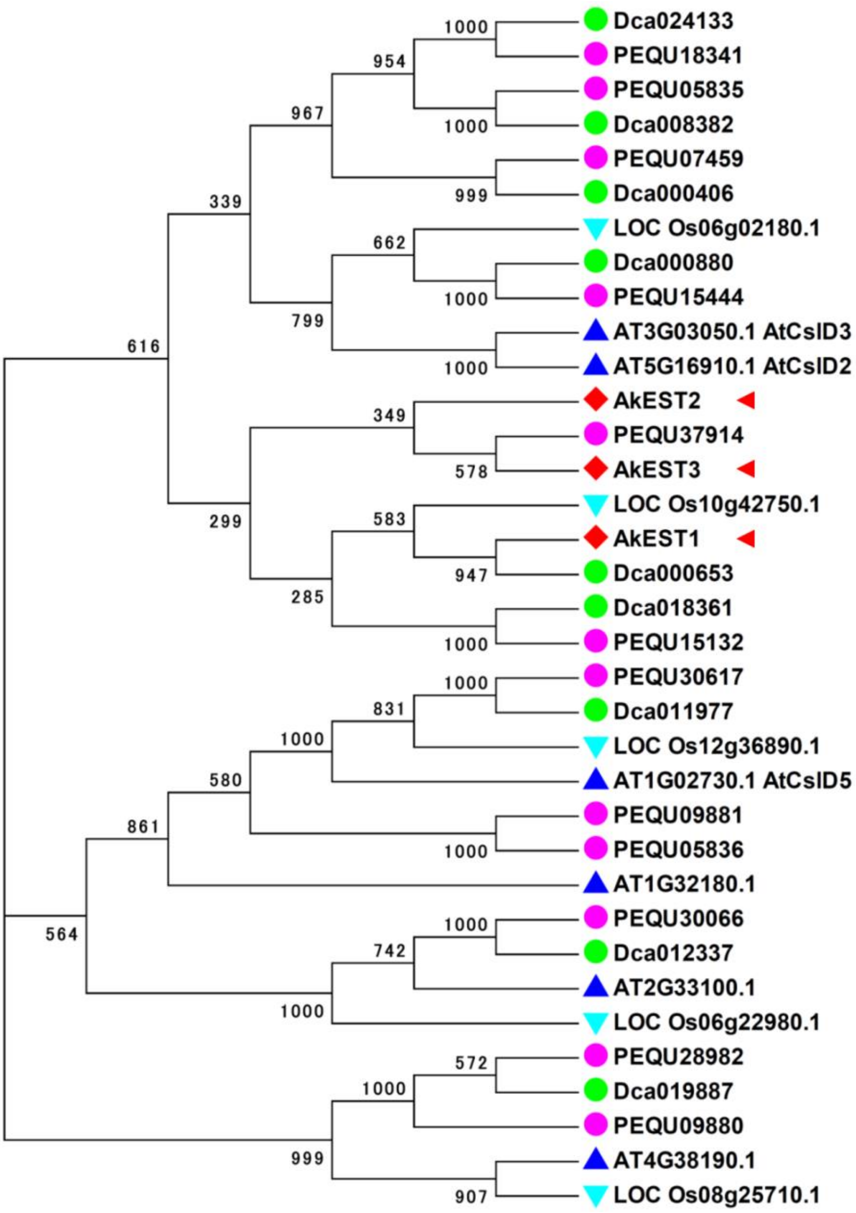

Supplementary Figure 14. The phylogenetic tree of *CslD*. The konjac putative *CslD* genes (EST clones) were included to search orthologues in the *D. catenatum* genome. The konjac sequences were labeled by red arrowheads. The light and deep blue triangles indicate rice (labeled LOC) and *A. thaliana* (AT), respectively. The green and pink dots represent *D. catenatum* (Dca) and *P. equestris* (PEQU). The sequences used here are longer than 150 amino acids. The konjac EST sequences were provided in **Supplementary Table 26**. Bootstrap values were shown on each branch.

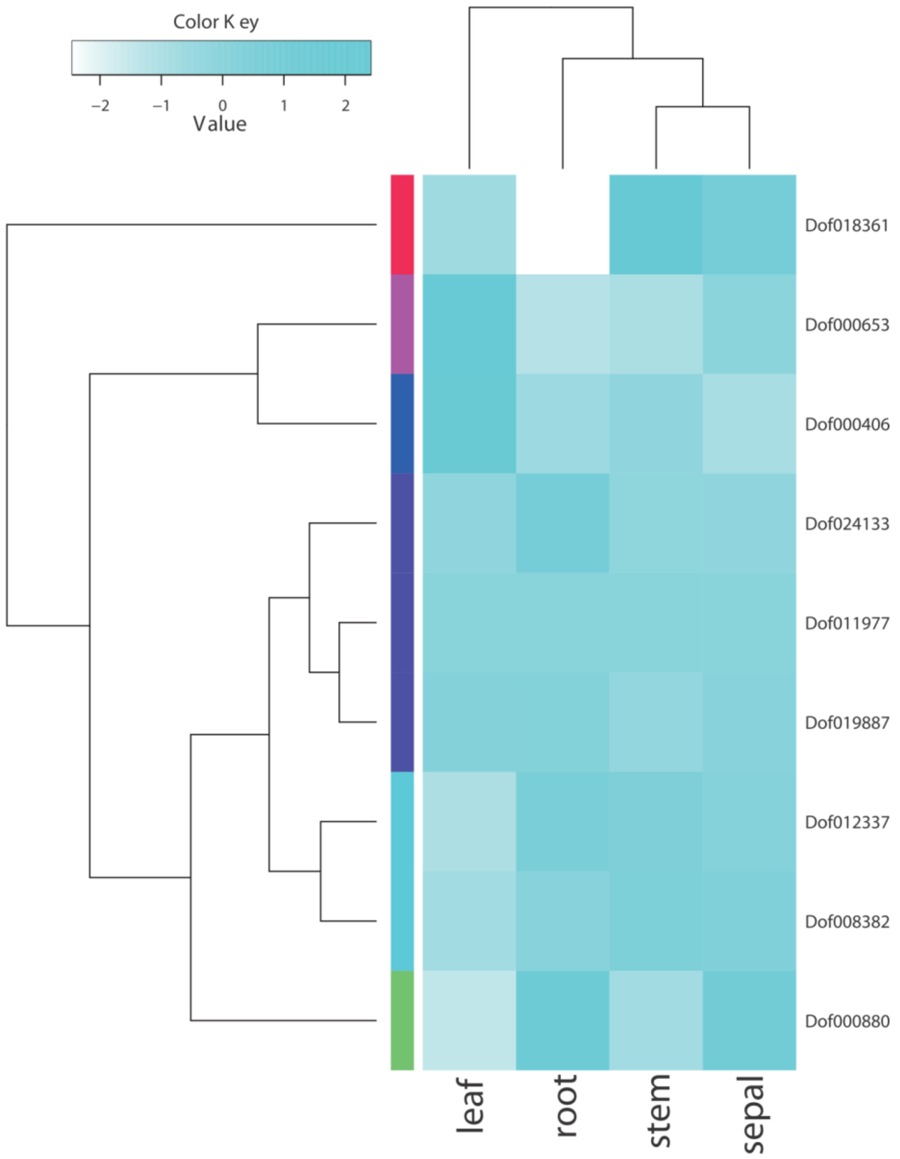

Supplementary Figure 15. Heat map showing the transcriptome expression of *CslD* genes in four tissues of *D. catenatum*. Genes shown in dark cyan are high-expressed, in contrast, light cyan represent that these genes are low-expressed.

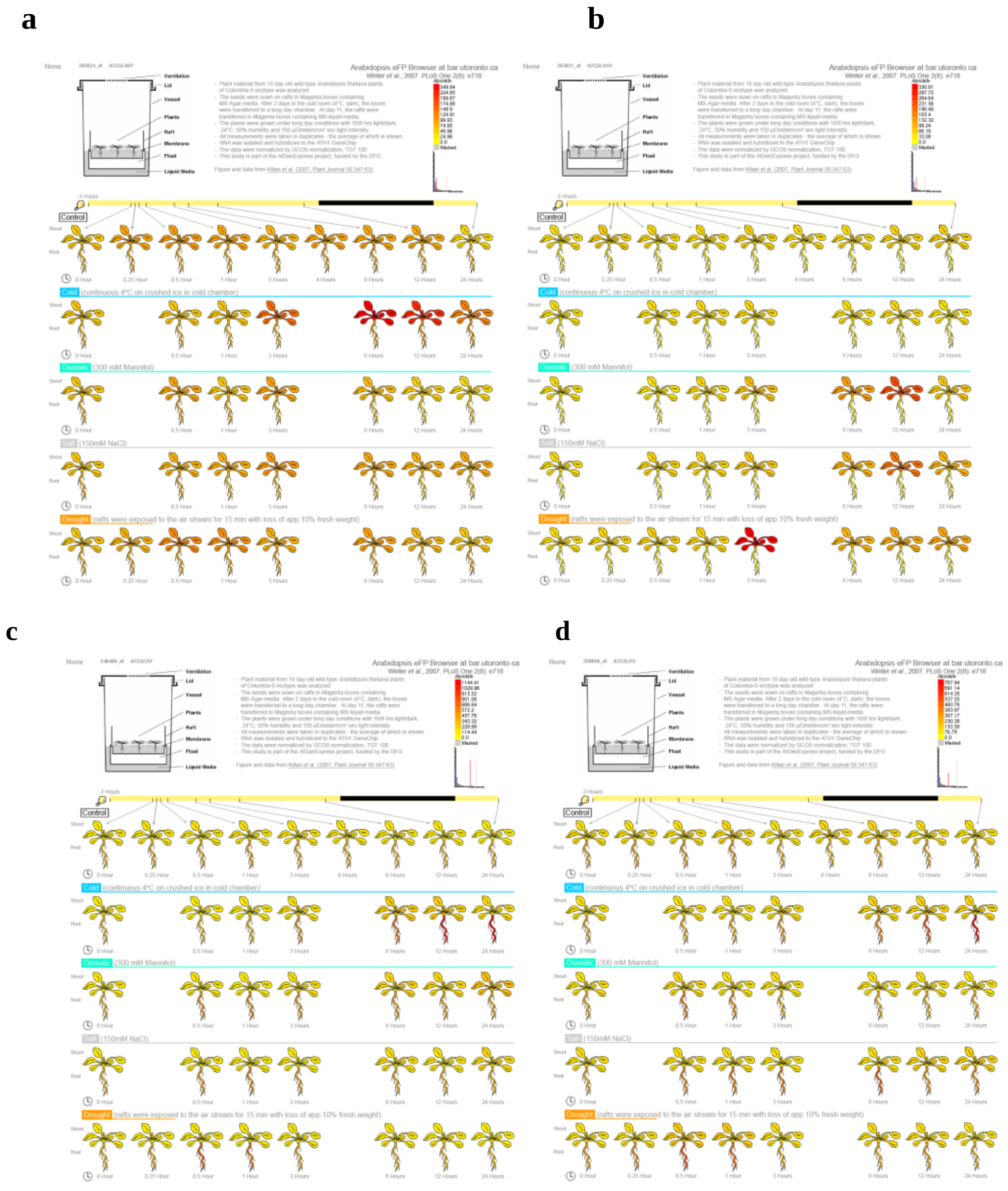

Supplementary Figure 16. Expression levels of Arabidopsis *CslA* and *CslD* genes in response to abiotic stresses. The microarray data were from the study performed by Kilian et al.[21]. The expression levels of *CslA7* (a), *CslA10* (b), *CslD2* (c) and *CslD3* (d) under cold, osmotic, salt and drought stresses were retrieved from the Arabidopsis eFP Browser[22] (http://bar.utoronto.ca/efp/cgi-bin/efpWeb.cgi). The color scale bar in each image shows the absolute expression levels for the individual gene. (We acknowledge the permission of Professor Nicholas Provart to use the images.)

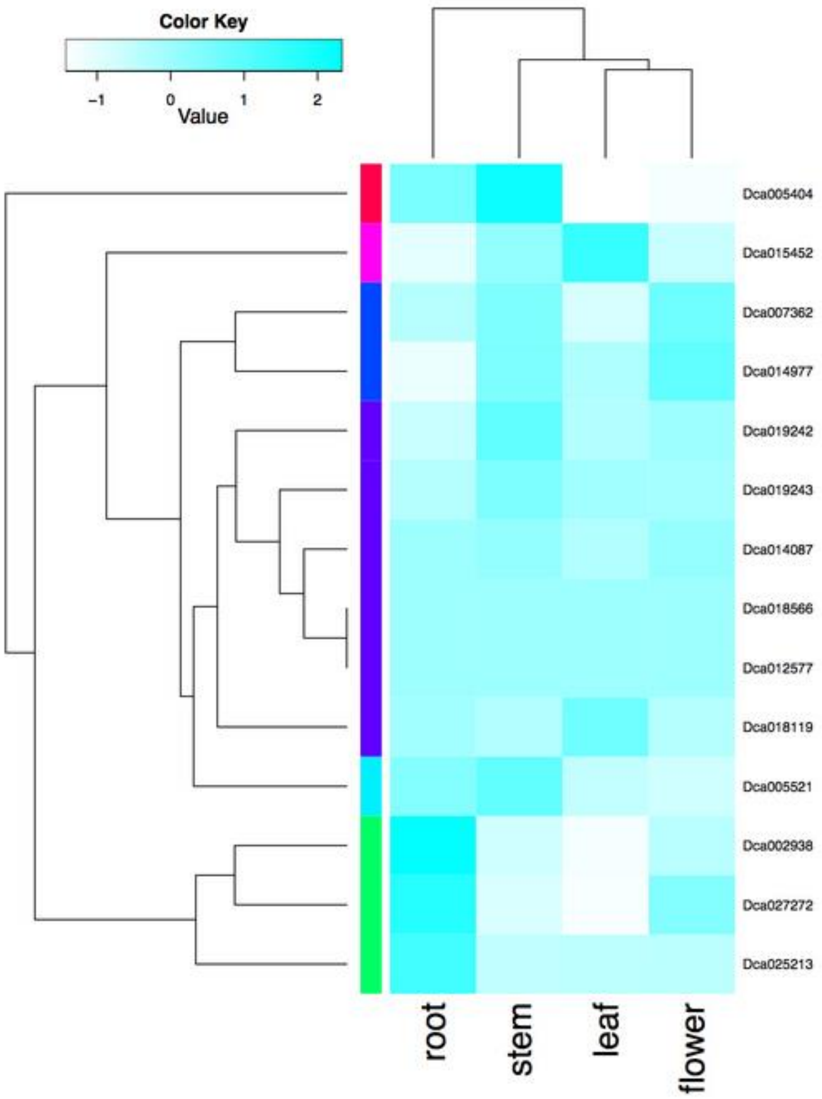

Supplementary Figure 17. Heat map showing the transcriptome expression of *GH5* genes in four tissues of *D. catenatum*. Genes shown in dark cyan are high-expressed, in contrast, light cyan represent that these genes are low-expressed.

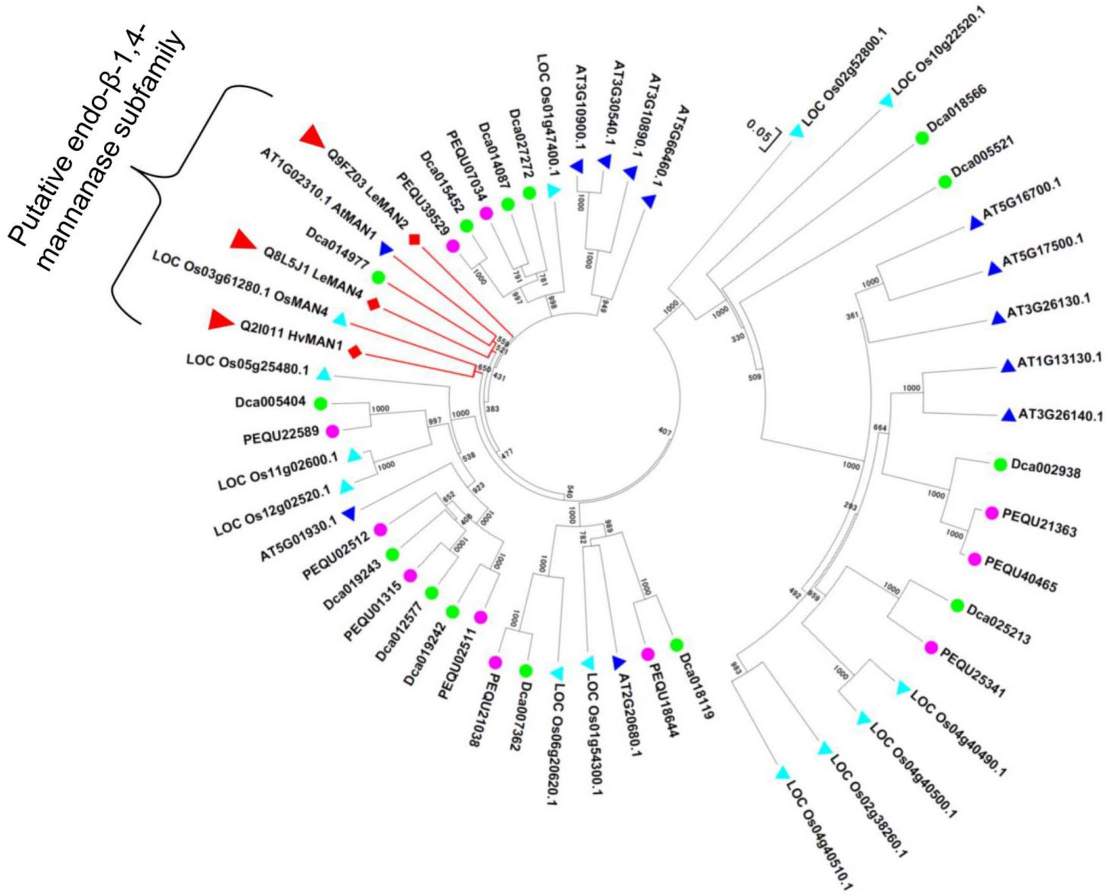

Supplementary Figure 18. The phylogenetic tree of *GH5* gene families. The *GH5* genes of barley (*HvMAN1*) and tomato (*LeMAN2* and *LeMAN4*) with proved mannanase activities were included and labeled by red arrowhead. The subclade containing these mannanase genes was indicated by red color. The light and deep blue triangles indicate rice (labeled LOC) and *A. thaliana* (AT), respectively. The green and pink dots represent *D. catenatum* (Dca) and *P. equestris* (PEQU). The sequences used here are longer than 200 amino acids. Bootstrap values were shown on each branch.

a

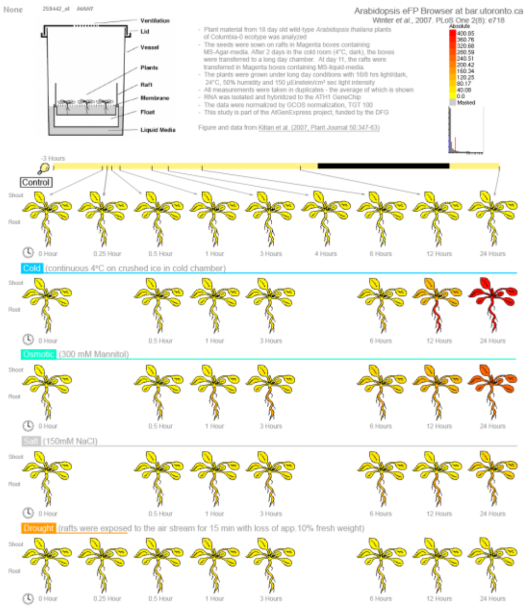

b

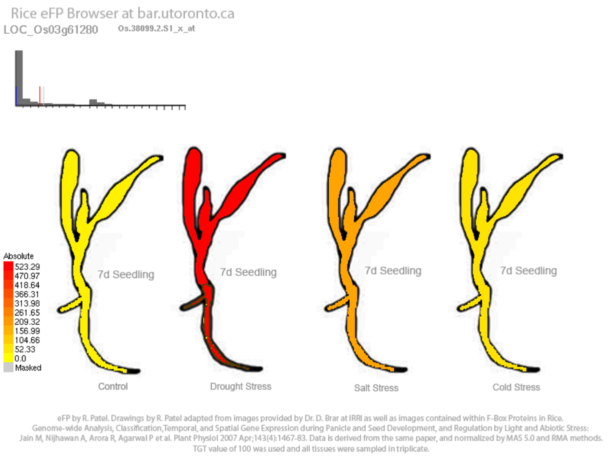

Supplementary Figure 19. Expression levels of Arabidopsis and rice *GH5* genes in response to abiotic stresses. The microarray data of Arabidopsis and rice were obtained from Kilian *et al*.[21] and Jain *et al*.[23], respectively. Expression levels of AtMAN1 (a) and Os03g61280 (b) under cold, osmotic, salt and drought stresses were retrieved from the Arabidopsis eFP Browser[22] (http://bar.utoronto.ca/efp/cgi-bin/efpWeb.cgi) and Rice eFP Browser (http://bar.utoronto.ca/efprice/cgi-bin/efpWeb.cgi). The color scale bar in each image shows the absolute expression level for each individual gene. (We acknowledge the permission of Professor Nicholas Provart to use the images.)

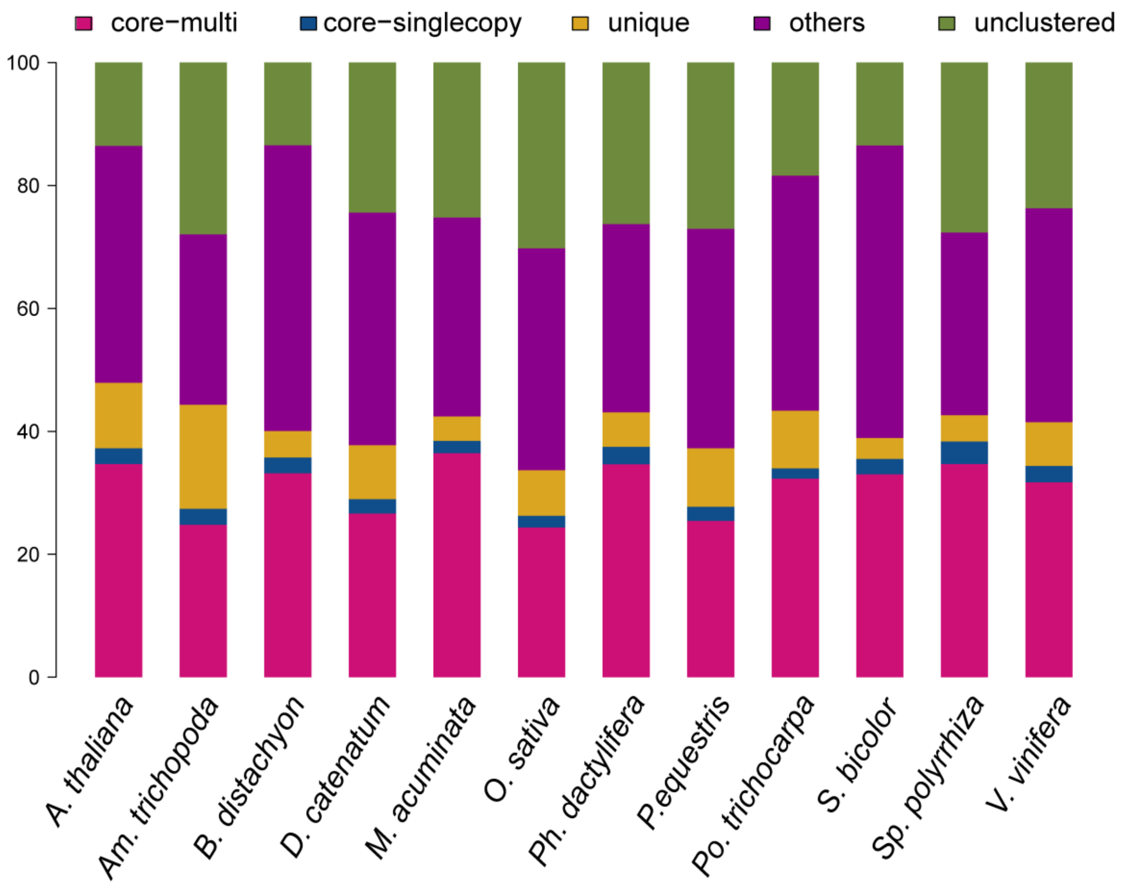

Supplementary Figure 20. Orthologous genes found in different plant species. **Core-multi**: genes that have orthologues in all other species and might have paralogues in other species. **Core-single copy**: genes that have orthologues in all other species and no other paralogues. Thus these genes are single copy in all species. **Unique**: Unique gene family for this particular species. **Other orthologues**: genes are not included in the other mentioned categories. **Unclustered genes**: genes that are unclustered into any family.

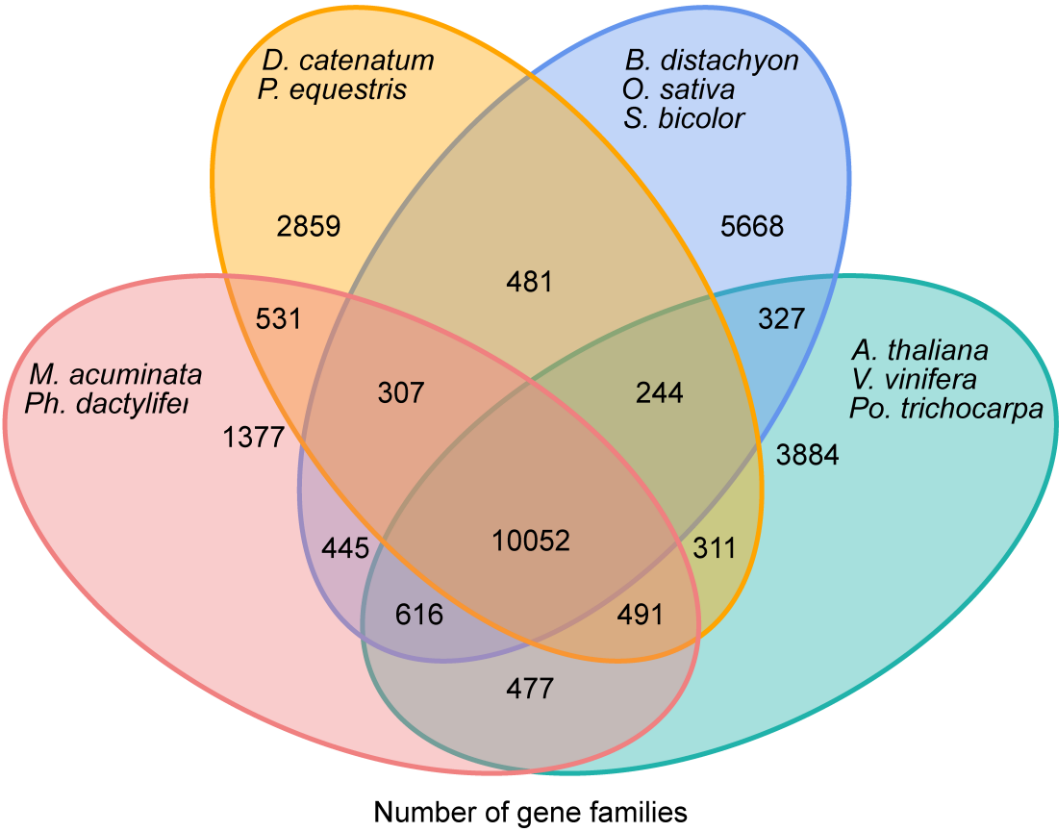

Supplementary Figure 21. Venn diagram showing unique and shared gene families among members of Orchidaceae, dicots and Poaceae, and *M. acuminata* and *Ph. dactylifera*. Numbers represent total shared or unique families. A total of 10,052 families were found in all categories. Comparison of the four categories revealed that 2,859 gene families were unique to Orchidaceae and 5,668 were unique to Poaceae. Note: when a gene family was found in only one species of a category, we considered the family to be specific to that category.

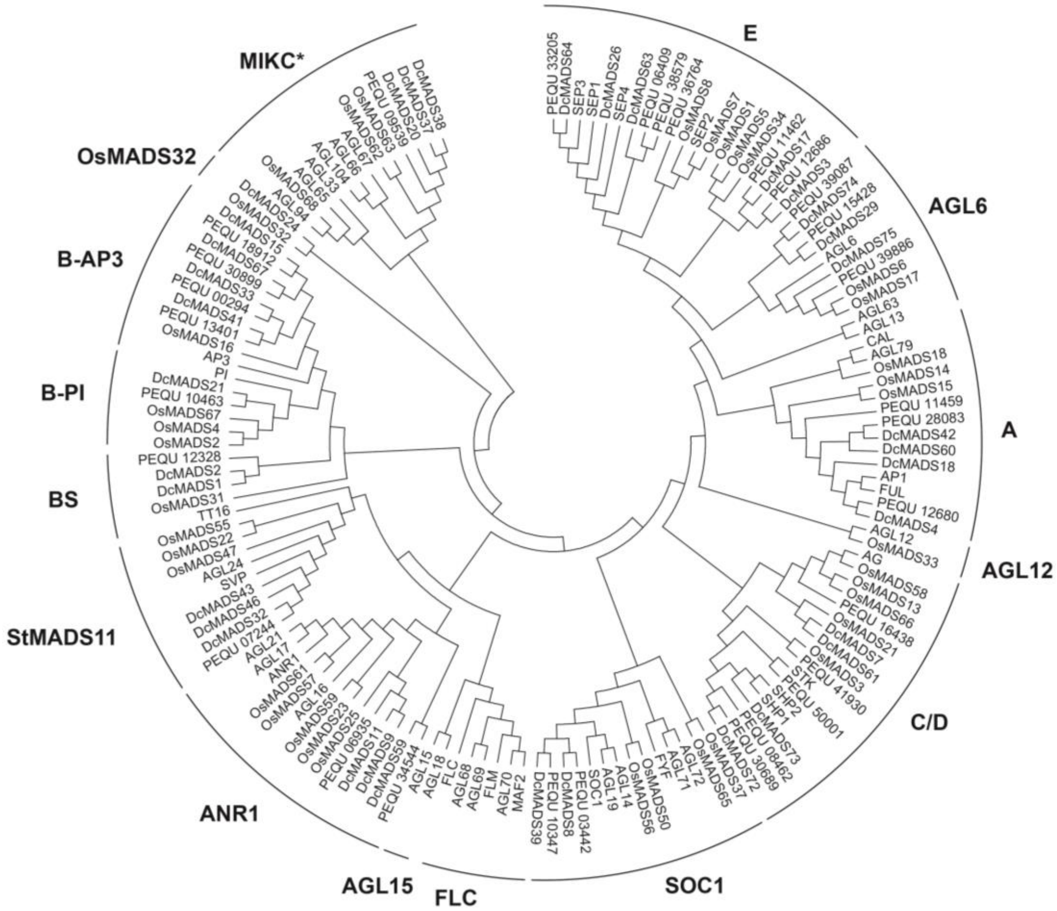

Supplementary Figure 22. Phylogenetic tree of Type II MADS-box genes from *O. sativa* (Os), *A. thaliana* (At), *P. equestris* (PEQU) and *D. catenatum* (Dc). Phylogenetic analysis indicates that most type II MADS-box genes have duplications in *D. catenatum*, except for those in the B-PI clade. Among these clades, *ANR1*, *StMADS11*, *MIKC**, and *Bs* contain more members than in *P. equestris.* Both *P. equestris* and *D. catenatum* lost members in the FLC, AGL12, and AGL15 clades.

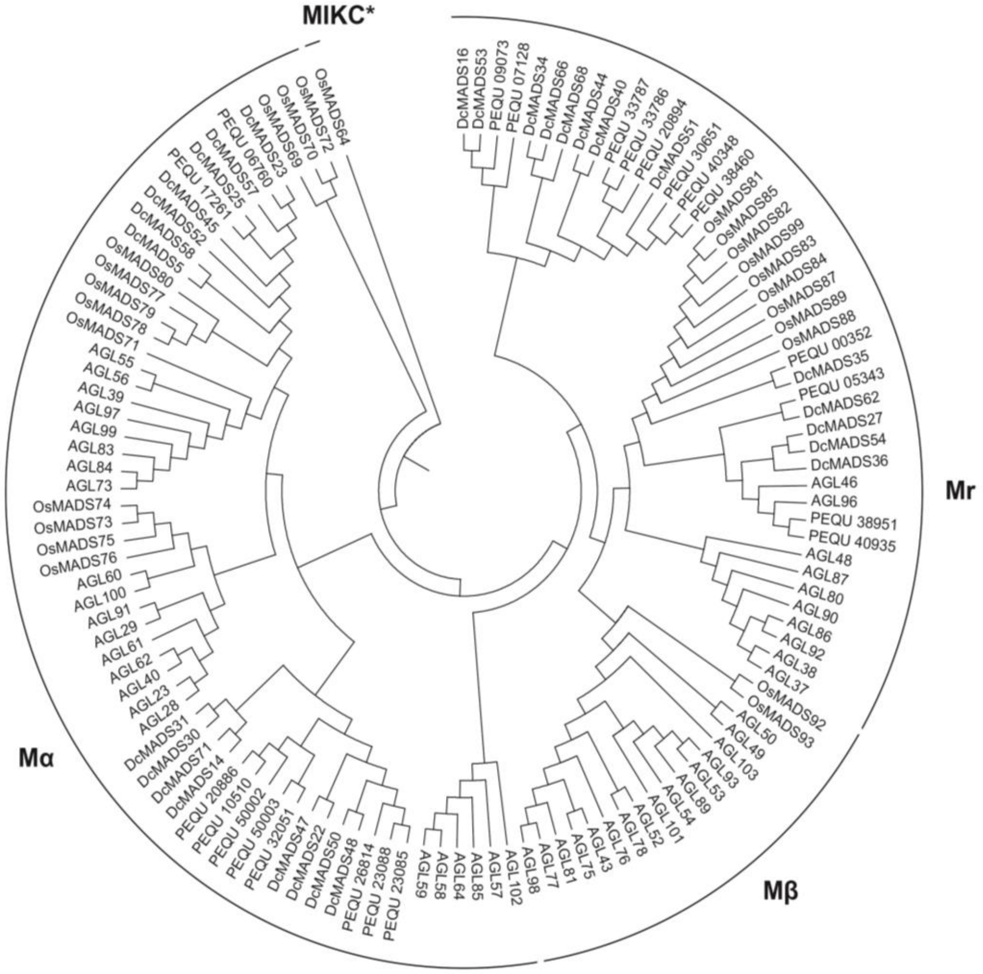

Supplementary Figure 23. Phylogenetic tree of Type I MADS-box genes from *O. sativa* (Os), *A. thaliana* (At), *P. equestris* (PEQU) and *D. catenatum* (Dc). The results indicate that *ANR1* (with three members), *StMADS11* (three members), *MIKC** (three members), and *Bs* (two members) contain more members than in *P. equestris* (two members in *ANR1* and one member in other three clades, respectively). Both P. equestris and *D. catenatum* lost members in the Mβ clades. 28 Type I MADS-box genes including 15 Mα and 13 Mγ members exist in the *D. catenatum* genome.

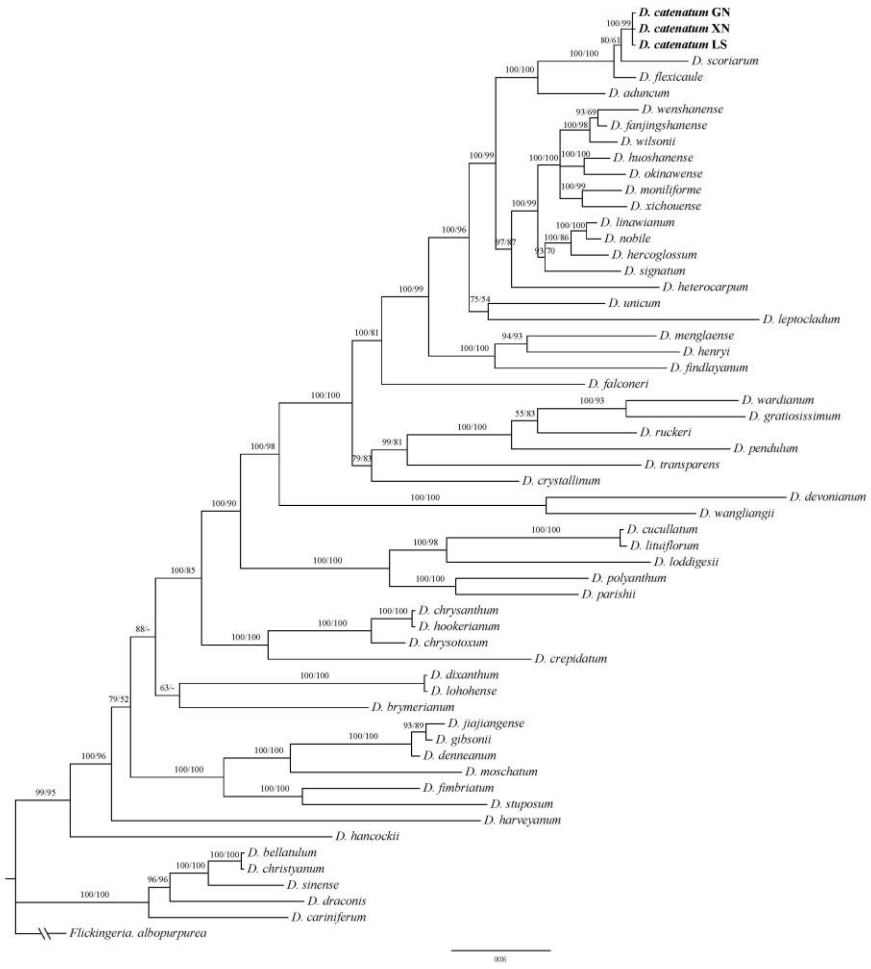

Supplementary Figure 24. Phylogenetic tree of *Dendrobium* based on nr ITS and plastid DNA. Phylogenetic analysis indicates that *D. catenatum* is sister to *D. scoriarum* and is an independent species. Numbers at nodes indicate Bayesian posterior probabilities and bootstrap values. “-” indicates that a node receives weak (<50) support in both ML and MP analyses.

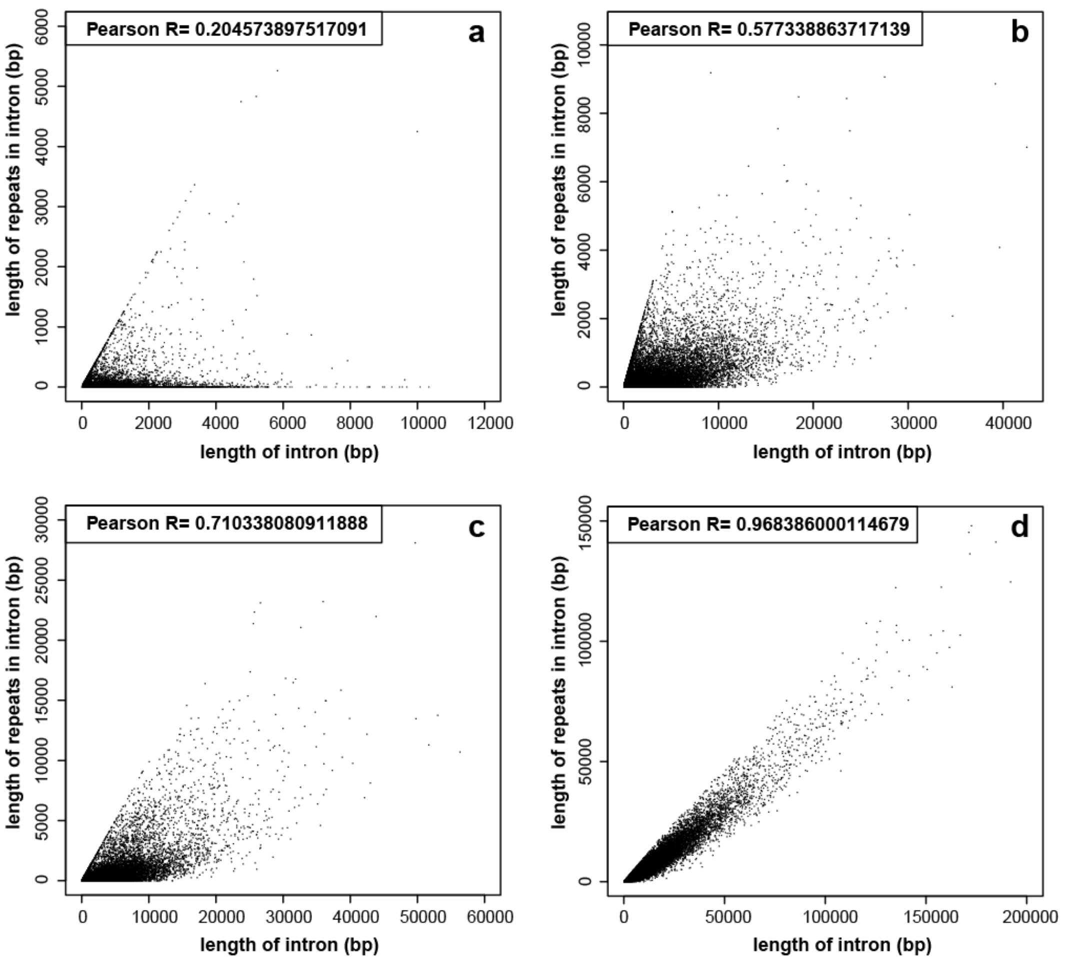

Supplementary Figure 25. Pearson's correlation analysis between intron length and intronic TE length for *Arabidopsis thaliana* **(a)**, *Phoenix dactylifera* **(b)**, *Musa acuminate* **(c)**, *Dendrobium catenatum* **(d)**. The results show that the longer the average intron, the stronger the correlation between intron length and intronic TE length, leading to an almost complete correlation in *D. catenatum*.

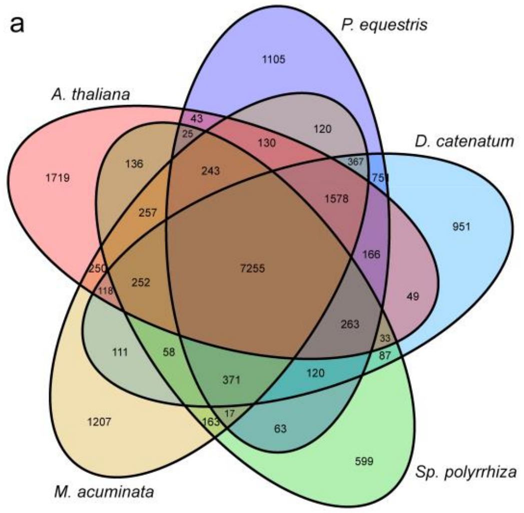

Supplementary Figure 26. Five-way Venn diagrams showing unique and shared gene families among different plant species. The results show the number of species-specific gene families. A. *D. catenatum*, *A. thaliana*, *P. equestris*, *M. acuminata* and *Sp. polyrrhiza*. B. *D. catenatum*, *A. thaliana*, *P. equestris*, *M. acuminata* and *O. sativa*.

Supplementary Tables

Supplementary Table 1. Summary of data generated for the *D. catenatum* genome sequencing with HiSeq 2000[a]

Insert size (bp)	Read type	Total data (Gb)	Sequence depth (X)
180	PE 100 bp	35.95	32.68
500	PE 90 bp	53.72	48.84
800	PE 100 bp	25.70	23.36
2000	MP 100 bp	30.95	28.14
5000	MP 100 bp	23.39	21.26
10,000	MP 90 bp	3.69	3.35
20,000	MP 90 bp	2.26	2.06
Total		175.66	159.69

[a]PE, paired-end; MP, mate-pair.

Supplementary Table 2. Summary of the *D. catenatum* genome assembly with Platanus

	Scaffold Length (bp)	Number	Contig Length (bp)	Number
Max length	2,592,627		288,536	
N10	1,139,106	70	87,703	847
N20	830,336	175	64,976	2,132
N30	636,735	315	51,190	3,801
N40	497,537	494	41,112	5,886
N50	391,462	723	33,094	8,479
N60	294,893	1,019	25,946	11,739
N70	210,351	1,424	19,133	16,018
N80	119,683	2,049	12,484	22,153
N90	15,394	3,978	5,113	33,556
Total_length	1,008,546,262		955,235,028	
number>=500 bp		72,903		105,732
number>=2000 bp		14,450		45,850
GC ratio	0.327		0.346	

Supplementary Table 3. CEGMA evaluation for the completeness of the *D. catenatum* genome assembly

	**Number**	**Completeness (%)**	**Total**	**Average**	**Orthology (%)**
Complete	231	93.15	346	1.50	32.47
Group 1	60	90.91	81	1.35	21.67
Group 2	52	92.86	78	1.50	32.69
Group 3	58	95.08	84	1.45	31.03
Group 4	61	93.85	103	1.69	44.26
Partial	242	97.58	405	1.67	39.26
Group 1	63	95.45	88	1.40	23.81
Group 2	54	96.43	92	1.70	40.74
Group 3	60	98.36	97	1.62	36.67
Group 4	65	100.00	128	1.97	55.38

Supplementary Table 4. Evaluation of the *D. catenatum* genome completeness using data set of RNA transcripts

Data set	Number	Total length	Covered by assembly (%)	With >90% sequence in one scaffold		With >50% sequence in one scaffold	
				Number	Percentage	Number	Percentage
>200 bp	32,571	40,232,920	93.43	26,109	80.16	29,428	90.35
>500 bp	24,054	37,553,793	94.31	19,655	81.71	22,606	93.98
>1,000 bp	16,529	31,953,475	94.70	13,438	81.30	15,671	94.80

Supplementary Table 5. Statistics for repetitive elements in the *D. catenatum* genome[a]

	RepBase TEs		TE Proteins		*De novo*		Combined TEs	
	**Length (bp)**	**% in Genome**	**Length (bp)**	**% in Genome**	**Length (bp)**	**% in Genome**	**Length (bp)**	**% in Genome**
DNA	14,119,342	1.20	12,286,880	1.05	60,007,381	5.11	71,186,132	6.06
LINE	33,207,011	2.83	76,796,121	6.53	140,798,500	11.98	169,998,018	14.46
SINE	80,012	0.01	0	0.00	1,392,811	0.12	1,457,837	0.12
LTR	105,535,338	8.98	130,748,856	11.12	526,202,517	44.77	537,358,505	45.72
Other	9,456	0.00	0	0.00	0	0.00	9,456	0.00
Unknown	62,119	0.01	0	0.00	29,518,345	2.51	29,579,608	2.52
Total	154,558,706	13.15	219,581,674	18.68	740,790,020	63.02	788,949,662	67.12

[a] Repbase transposable elements (TEs): the result of RepeatMasker based on Repbase; TE proteins: the result of RepeatProteinMask based on Repbase; *De novo*: repeats found with the *de novo* library; Combined: combined results of the Repbase TEs, TE proteins and *De novo* repeats.

Supplementary Table 6. Gene models supported by differing evidence types

	>=20% Overlap		>=50% Overlap		>=80% Overlap	
	**No.**	**Ratio (%)**	**No.**	**Ratio (%)**	**No.**	**Ratio (%)**
De novo (single)*	2,350	8.13	3,616	12.51	5,464	18.9
De novo (more)**	820	2.84	753	2.6	712	2.46
Homologue (single)	1,273	4.4	1,095	3.79	969	3.35
Homologue (more)	515	1.78	598	2.07	781	2.7
RNA	1,288	4.46	1,562	5.4	2,804	9.7
D+H	7,043	24.36	5,925	20.49	3,879	13.42
D+R	1,215	4.2	1,644	5.69	2,982	10.31
H+R	664	2.3	1,168	4.04	2,375	8.22
D+H+R	13,638	47.17	12,062	41.72	7,743	26.78

*(single) indicates that the genes were predicted by only one annotation method.
**(more) indicates that the genes were annotated by at least two methods.

Supplementary Table 7. Statistics of gene element length for seven sequenced plant species

**Species**	**Protein-coding gene number**	**Average gene length (bp)**	**Average CDS length (bp)**	**Average exon per gene**	**Average exon length (bp)**	**Average intron length (bp)**
D. catenatum	28,910	10,192.33	1002.25	4.13	242.77	2575.18
P. equestris	29,431	9644.63	897.62	3.93	228.27	2922.24
Z. mays	38,510	3965.59	1101.62	4.54	242.75	637.83
S. bicolor	27,159	2942.10	1261.01	4.85	259.90	436.44
O. sativa	40,745	2439.30	1117.21	4.18	266.99	415.17
Phy. heterocycla	31,987	4244.98	1210.22	5.28	229.03	440.95
A. thaliana	26,637	1909.57	1242.78	5.23	237.50	157.54

Supplementary Table 8. Statistics for noncoding RNAs in *D. catenatum*

Type of noncoding DNA		Copy number	Average length (bp)	Total length (bp)	% of genome
miRNA		49	125.02	6,126	0.00052
tRNA		310	75.35	23,357	0.00199
rRNA	rRNA	248	235.25	58,342	0.00496
	18S	107	390.24	41,756	0.00355
	28S	26	146.85	3,818	0.00033
	5.8S	14	147.57	2,066	0.00018
	5S	101	105.96	10,702	0.00091
snRNA	snRNA	144	117.74	16,955	0.00144
	CD-box	34	108.65	3,694	0.00031
	HACA-	1	166.00	166	0.00001
	splicing	109	120.14	13,095	0.00111
	scaRNA	0	0.00	0	0.00000

Supplementary Table 9. GO term enrichment results of significantly expanded gene families in the *D. catenatum* lineage

GO ID	GO Term	Class	*P*-value	Adjusted *P*-value
GO:0015074	DNA integration	BP	2.03E-216	3.76E-214
GO:0006259	DNA metabolic process	BP	2.64E-181	4.89E-179
GO:0090304	Nucleic acid metabolic process	BP	3.13E-92	5.79E-90
GO:0003964	RNA-directed DNA polymerase activity	MF	1.06E-70	1.96E-68
GO:0006278	RNA-dependent DNA replication	BP	1.06E-70	1.96E-68
GO:0006952	Defence response	BP	3.48E-57	6.43E-55
GO:0043531	ADP binding	MF	1.92E-55	3.56E-53
GO:0003723	RNA binding	MF	9.04E-40	1.67E-37
GO:0043170	Macromolecule metabolic process	BP	1.90E-38	3.51E-36
GO:0044260	Cellular macromolecule metabolic process	BP	2.49E-37	4.60E-35
GO:0006950	Response to stress	BP	5.26E-28	9.74E-26
GO:0004523	Ribonuclease H activity	MF	4.05E-25	7.49E-23
GO:0003676	Nucleic acid binding	MF	5.27E-23	9.76E-21
GO:0044238	Primary metabolic process	BP	5.45E-23	1.01E-20
GO:0016772	Transferase activity, transferring phosphorus-containing groups	MF	1.13E-13	2.10E-11
GO:0004190	Aspartic-type endopeptidase activity	MF	1.23E-12	2.27E-10
GO:0046983	Protein dimerisation activity	MF	1.06E-11	1.96E-09
GO:0034645	Cellular macromolecule biosynthetic process	BP	3.07E-10	5.68E-08
GO:0008152	Metabolic process	BP	5.77E-10	1.07E-07
GO:0008270	Zinc ion binding	MF	1.67E-06	0.00030842
GO:0016740	Transferase activity	MF	7.92E-06	0.00146498
GO:0016788	Hydrolase activity, acting on ester bonds	MF	5.57E-05	0.01029978
GO:0009616	Virus-induced gene silencing	BP	0.0001058	0.01958041
GO:0005488	Binding	MF	0.0001069	0.01976832

Supplementary Table 10. KEGG pathway enrichment results for SNP-related genes

Map ID	Map title	*P*-value	Adjusted *P*-value
map01110	Biosynthesis of secondary metabolites	3.34E-08	4.27E-06
map04075	Plant hormone signal transduction	7.00E-07	8.97E-05
map01100	Metabolic pathways	2.03E-05	0.002604
map00943	Isoflavonoid biosynthesis	0.000167	0.021423

Supplementary Table 11. GO term enrichment results for SNP-related genes

GO_ID	GO_Term	Class	P value	Adjusted P value	GO level
GO:0005488	Binding	MF	1.72E-34	4.52E-31	2
GO:0003824	Catalytic activity	MF	1.31E-33	3.42E-30	2
GO:0005515	Protein binding	MF	1.94E-24	5.08E-21	3
GO:0008152	Metabolic process	BP	6.34E-18	1.66E-14	2
GO:0030554	Adenyl nucleotide binding	MF	4.04E-16	1.06E-12	6
GO:0032559	Adenyl ribonucleotide binding	MF	1.42E-15	3.72E-12	7
GO:0005524	ATP binding	MF	3.82E-15	1.00E-11	8
GO:0016740	Transferase activity	MF	4.68E-15	1.23E-11	3
GO:0004713	Protein tyrosine kinase activity	MF	4.49E-13	1.18E-09	7
GO:0016773	Phosphotransferase activity, alcohol group as acceptor	MF	4.49E-12	1.18E-08	5
GO:0000166	Nucleotide binding	MF	1.42E-11	3.72E-08	4
GO:0036094	Small molecule binding	MF	1.62E-11	4.25E-08	3
GO:0046914	Transition metal ion binding	MF	2.45E-11	6.41E-08	6
GO:0016301	Kinase activity	MF	2.96E-11	7.75E-08	5
GO:0016310	Phosphorylation	BP	4.58E-11	1.20E-07	6
GO:0006468	Protein phosphorylation	BP	5.36E-11	1.41E-07	6
GO:0004672	Protein kinase activity	MF	5.89E-11	1.54E-07	6
GO:0017076	Purine nucleotide binding	MF	2.07E-10	5.42E-07	5
GO:0006796	Phosphate-containing compound metabolic process	BP	5.28E-10	1.38E-06	5
GO:0032555	Purine ribonucleotide binding	MF	5.36E-10	1.40E-06	6
GO:0016787	Hydrolase activity	MF	5.76E-10	1.51E-06	3
GO:0043412	Macromolecule modification	BP	6.15E-10	1.61E-06	4
GO:0006464	Protein modification process	BP	6.47E-10	1.69E-06	5
GO:0055114	Oxidation-reduction process	BP	1.13E-09	2.97E-06	3
GO:0035639	Purine ribonucleoside triphosphate binding	MF	1.87E-09	4.91E-06	7
GO:0046872	Metal ion binding	MF	3.71E-09	9.73E-06	5
GO:0043169	Cation binding	MF	4.47E-09	1.17E-05	4
GO:0016705	Oxidoreductase activity, acting on paired donors, with incorporation or reduction of molecular oxygen	MF	6.20E-09	1.62E-05	4
GO:0016772	Transferase activity, transferring phosphorus-containing groups	MF	1.72E-08	4.51E-05	4
GO:0016491	Oxidoreductase activity	MF	2.45E-08	6.41E-05	3
GO:0005506	Iron ion binding	MF	4.39E-08	0.000115	7
GO:0044238	Primary metabolic process	BP	1.52E-07	0.000397	3

GO:0020037	Heme binding	MF	4.55E-07	0.001193	4
GO:0046906	Tetrapyrrole binding	MF	5.91E-07	0.001549	3
GO:0043086	Negative regulation of catalytic activity	BP	6.66E-07	0.001745	5
GO:0042802	Identical protein binding	MF	9.41E-07	0.002466	4
GO:0016798	Hydrolase activity, acting on glycosyl bonds	MF	1.25E-06	0.003269	4
GO:0043170	Macromolecule metabolic process	BP	1.34E-06	0.003503	3
GO:0019222	Regulation of metabolic process	BP	1.53E-06	0.003998	3
GO:0008017	Microtubule binding	MF	2.18E-06	0.005704	6
GO:0006950	Response to stress	BP	2.61E-06	0.006825	3
GO:0070011	Peptidase activity, acting on L-amino acid peptides	MF	7.18E-06	0.018805	5
GO:0006508	Proteolysis	BP	8.11E-06	0.021247	5
GO:0008233	Peptidase activity	MF	8.49E-06	0.022255	4
GO:0004553	Hydrolase activity, hydrolysing O-glycosyl compounds	MF	8.61E-06	0.02256	5

Supplementary Table 12. Tandem duplicated genes in *D. catenatum* shown as gene pairs

See separate Excel file.

Supplementary Table 13. Tandem duplicated genes in *P. equestris* shown as gene pairs

See separate Excel file.

Supplementary Table 14. List of the resistant genes of *D. catenatum* and *P. equestris*

	D. catenatum	*P. equestris*
CC_NBS	14	7
CC_NBS_LRR	13	8
NBS_LRR	47	17
NBS	83	47

Supplementary Table 15. Summary of orthologous gene families in 12 sequenced plant species

Species[a]	Genes	Unclustered genes	Genes in families	Family number	Unique families	Unique families genes	Common families	Common families genes	Single copy	Average genes per family
A. thaliana	26,637	3,614	23,023	12,517	741	2,844	5,238	9,921	677	1.839
Am. trichopoda	25,933	7,247	18,686	12,149	996	4,388	5,238	7,108	677	1.538
B. distachyon	26,415	3,555	22,860	14,987	386	1,133	5,238	9,447	677	1.525
D. catenatum	28,910	7,053	21,857	13,530	629	2,533	5,238	8,381	677	1.615
M. acuminata	34,241	8,632	25,609	12,497	540	1,361	5238	13,165	677	2.049
O. sativa	35,402	10,699	24,703	16,056	972	2,616	5,238	9,305	677	1.539
Ph. dactylifera	23,890	6,280	17,610	10,660	428	1,339	5,238	8,958	677	1.652
P. equestris	29,431	7,955	21,476	13,617	621	2,799	5,238	8,167	677	1.577
Po. trichocarpa	40,984	7,544	33,440	14,122	1,200	3,843	5,238	13,929	677	2.368
S. bicolor	27,160	3,661	23,499	15,306	338	929	5,238	9,645	677	1.535
Sp. polyrrhiza	18,357	5,076	13,281	9,942	260	781	5,238	7,044	677	1.336
V. vinifera	25,328	6,007	19,321	12,551	617	1,797	5,238	8,711	677	1.539

[a], unique families, families only contained in one species.

Supplementary Table 16. List of the putative genes involved in GM/GGM synthesis and hydrolysis in the *D. catenatum* genome

Function	Gene ID	Name	Protein length (aa)	Phylogenetic classification	Signal peptide	TMD	Sequence type
Nucleotide-sugar transport	Dca005389	DcaNST/TPT1	349	Ia: URGT[*1]	No	10	Full
	Dca012954	DcaNST/TPT2	340	Ia: URGT	No	10	Full
	Dca021203	DcaNST/TPT3	348	Ia: URGT	No	10	Full
	Dca005907	DcaNST/TPT4	332	Ib: URGT	No	10	Full
	Dca016070	DcaNST/TPT5	335	Ib: URGT	No	10	Full
	Dca017392	DcaNST/TPT6	349	Ic: unknown	No	10	Full
	Dca024394	DcaNST/TPT7	352	Ic: unknown	No	10	Full
	Dca002549	DcaNST/TPT8	343	II: UTR[*2]	No	8	Full
	Dca010609	DcaNST/TPT9	332	II: UTR	No	9	Full
	Dca005243	DcaNST/TPT10	403	III: GONST[*3]	No	9	Full
	Dca001640	DcaNST/TPT11	378	III: GONST	No	10	Full
	Dca006839	DcaNST/TPT12	404	III: UTR	No	9	Full
	Dca008480	DcaNST/TPT13	430	III: GONST	No	10	Full
	Dca017354	DcaNST/TPT14	350	III: GONST	No	10	Full
	Dca006012	DcaNST/TPT15	381	V: unknown	No	10	Full
	Dca011003	DcaNST/TPT16	339	V: GONST	No	9	Full
	Dca019884	DcaNST/TPT17	356	V: unknown	No	10	Full
	Dca013292	DcaNST/TPT18	356	VI: unknown	No	10	Full
	Dca015521	DcaNST/TPT19	318	VI: unknown	No	10	Full
GM/GGM backbone synthesis	Dca005598	DcaCslA1	536	GT2 family CslA	No	5	Full
	Dca006365	DcaCslA2	499	GT2 family CslA	Yes	5	Full
	Dca006366	DcaCslA3	522	GT2 family CslA	No	5	Full
	Dca006368	DcaCslA4	588	GT2 family CslA	Yes	5	Full
	Dca007032	DcaCslA5	556	GT2 family CslA	No	5	Full
	Dca007033	DcaCslA6	302	GT2 family CslA	No	4	Fragment
	Dca007034	DcaCslA7	564	GT2 family CslA	No	6	Full
	Dca007035	DcaCslA8	599	GT2 family CslA	Yes	5	Full
	Dca008185	DcaCslA9	528	GT2 family CslA	No	5	Full
	Dca013434	DcaCslA10	550	GT2 family CslA	No	5	Full
	Dca013437	DcaCslA11	549	GT2 family CslA	No	5	Full
	Dca018319	DcaCslA12	227	GT2 family CslA	No	0	Fragment
	Dca022357	DcaCslA13	543	GT2 family CslA	No	5	Full
	Dca000406	DcaCslD1	581	GT2 family CslD	No	6	Fragment
	Dca000653	DcaCslD2	215	GT2 family CslD	No	0	Fragment
	Dca000880	DcaCslD3	1153	GT2 family CslD	No	8	Full
	Dca008382	DcaCslD4	1215	GT2 family CslD	No	7	Full
	Dca011977	DcaCslD5	1150	GT2 family CslD	Yes	7	Full

	Dca012337	DcaCslD6	980	GT2 family CslD	No	7	Fragment
	Dca018361	DcaCslD7	383	GT2 family CslD	No	0	Fragment
	Dca019887	DcaCslD8	1172	GT2 family CslD	No	7	Full
	Dca024133	DcaCslD9	1140	GT2 family CslD	No	7	Full
Mannan- synthesis related	Dca004648	Dca MSR1	413	O-FucT[*4]	No	1	Full
Acetylation	Dca002655	DcaRWA1	546	Acetyl-CoA T[*5]	No	14	Full
	Dca004270	DcaRWA2	545	Acetyl-CoA T	No	14	Full
	Dca014073	DcaRWA3	545	Acetyl-CoA T	No	14	Full
GM/GGM endolytic hydrolysis	Dca005404	DcaGH5-1	469	Mannase	No	1	Full
	Dca007362	DcaGH5-2	432	Mannase	Yes	0	Full
	Dca012577	DcaGH5-3	461	Mannase	Yes	0	Full
	Dca014087	DcaGH5-4	421	Mannase	Yes	0	Full
	Dca014977	DcaGH5-5	411	Mannase	Yes	0	Full
	Dca015452	DcaGH5-6	429	Mannase	Yes	0	Full
	Dca018119	DcaGH5-7	445	Mannase	No	1	Full
	Dca019242	DcaGH5-8	442	Mannase	Yes	0	Full
	Dca019243	DcaGH5-9	439	Mannase	Yes	0	Full
	Dca027272	DcaGH5-10	236	Mannase	No	0	Fragment
	Dca002938	DcaGH5-11	549	Cellulase	Yes	0	Full
	Dca025213	DcaGH5-12	524	Cellulase	Yes	0	Full
	Dca005521	DcaGH5-13	300	Unknown	No	0	Fragment
	Dca018566	DcaGH5-14	426	Unknown	No	1	Fragment

The prediction of signal peptide and transmembrane domain (TMD) was performed by Phobius (http://phobius. sbc.su.se/). The Nucleotide sugar transporter/triosephosphate translocator family (NST/TPT) genes were divided into six groups based on the classification by Rautengarten et al.[24]. URGT: UDP-rhamnose/UDP-galactose transporter[*1]. UTR: UDP-galactose/UDP-glucose transporter[*2]. GONST: Golgi-localised nucleotide-sugar transporter[*3]. GDP-fucose protein O-fucosyltransferase[*4]. Acetyl-CoA T: acetyl-CoA transporter[*5].

Supplementary Table 17. List of the 75 MADS-box genes identified in *D. catenatum*

Gene ID	Name	ORF (bp)	Protein length (aa)	Type	Subfamily	Pseudogene (✓)
Dca006090	DcMADS1	291	96	MIKC^c	Bs	
Dca006092	DcMADS2	687	228	MIKC^c	Bs	
Dca021261	DcMADS3	741	246	MIKC^c	E	
Dca022092	DcMADS4	762	253	MIKC^c	SQUA	
Dca014289	DcMADS5	792	263	Type I	Mα	
Dca012372	DcMADS6	447	148	MIKC^c	C/D	✓
Dca012374	DcMADS7	255	84	MIKC^c	C/D	
Dca019419	DcMADS8	753	250	MIKC^c	SOC1	
Dca002134	DcMADS9	579	192	MIKC^c	ANR1	
Dca002135	DcMADS10	624	207	MIKC^c	ANR1	✓
Dca002136	DcMADS11	951	316	MIKC^c	ANR1	
Dca023400	DcMADS12	228	75	MIKC^c	Bs	✓
Dca014821	DcMADS13	186	61	MIKC^c	AGL6	✓
Dca022131	DcMADS14	555	184	Type I	Mα	
Dca005941	DcMADS15	675	224	MIKC^c	B_AP3	
Dca002732	DcMADS16	1059	352	Type I	Mγ	
Dca003023	DcMADS17	735	244	MIKC^c	E	
Dca003026	DcMADS18	687	228	MIKC^c	SQUA	
Dca003027	DcMADS19	852	283	MIKC^c	SQUA	✓
Dca028043	DcMADS20	459	152	MIKC*		
Dca000690	DcMADS21	633	210	MIKC^c	B_PI	
Dca027568	DcMADS22	621	206	Type I	Mα	
Dca014095	DcMADS23	708	235	Type I	Mα	
Dca018107	DcMADS24	579	192	MIKC^c	OsMADS32	
Dca027941	DcMADS25	678	225	Type I	Mα	
Dca011228	DcMADS26	354	117	MIKC^c	E	
Dca003978	DcMADS27	750	249	Type I	Mγ	
Dca000888	DcMADS28	561	186	MIKC^c	AGL6	✓
Dca000889	DcMADS29	483	160	MIKC^c	AGL6	
Dca019204	DcMADS30	1191	396	Type I	Mα	
Dca019205	DcMADS31	726	241	Type I	Mα	
Dca013716	DcMADS32	687	228	MIKC^c	StMADS11	
Dca018192	DcMADS33	657	218	MIKC^c	B_AP3	
Dca028556	DcMADS34	594	197	Type I	Mγ	
Dca022563	DcMADS35	771	256	Type I	Mγ	
Dca018025	DcMADS36	747	248	Type I	Mγ	
Dca016199	DcMADS37	840	279	MIKC*		
Dca016201	DcMADS38	930	309	MIKC*		

Dca014620	DcMADS39	642	213	MIKC^c	SOC1	
Dca022549	DcMADS40	957	318	Type I	Mγ	
Dca019113	DcMADS41	642	213	MIKC^c	B_AP3	
Dca017703	DcMADS42	366	121	MIKC^c	SQUA	
Dca012304	DcMADS43	687	228	MIKC^c	StMADS11	
Dca000568	DcMADS44	534	177	Type I	Mγ	
Dca006211	DcMADS45	693	230	Type I	Mα	
Dca024877	DcMADS46	690	229	MIKC^c	StMADS11	
Dca021832	DcMADS47	447	148	Type I	Mα	
Dca021833	DcMADS48	435	144	Type I	Mα	
Dca021834	DcMADS49	159	52	Type I	Mα	✓
Dca021835	DcMADS50	306	101	Type I	Mα	
Dca025806	DcMADS51	891	296	Type I	Mγ	
Dca007552	DcMADS52	348	115	Type I	Mα	
Dca000972	DcMADS53	858	285	Type I	Mγ	
Dca007676	DcMADS54	903	300	Type I	Mγ	
Dca007911	DcMADS55	228	75	MIKC^c	AGL6	✓
Dca007912	DcMADS56	831	276	MIKC^c	SOC1	✓
Dca006775	DcMADS57	708	235	Type I	Mα	
Dca006778	DcMADS58	405	134	Type I	Mα	
Dca018003	DcMADS59	696	231	MIKC^c	ANR1	
Dca002059	DcMADS60	738	245	MIKC^c	SQUA	
Dca003078	DcMADS61	672	223	MIKC^c	C/D	
Dca005316	DcMADS62	903	300	Type I	Mγ	
Dca018065	DcMADS63	732	243	MIKC^c	E	
Dca016730	DcMADS64	732	243	MIKC^c	E	
Dca016731	DcMADS65	213	70	MIKC^c	E	✓
Dca019717	DcMADS66	594	197	Type I	Mγ	
Dca012472	DcMADS67	669	222	MIKC^c	B_AP3	
Dca025329	DcMADS68	321	106	Type I	Mγ	
Dca027465	DcMADS69	186	61	MIKC*		✓
Dca027466	DcMADS70	219	72	MIKC*		✓
Dca016433	DcMADS71	534	177	Type I	Mα	
Dca100001	DcMADS72	702	233	MIKC^c	C/D	
Dca100002	DcMADS73	705	234	MIKC^c	C/D	
Dca100003	DcMADS74	720	239	MIKC^c	AGL6	
Dca100004	DcMADS75	750	249	MIKC^c	AGL6	

✓, represents this gene has been confirmed to be a pseudogene.

Supplementary Table 18. Summary of the *D. catenatum* genome assembly obtained with SOAPdenovo2

	Scaffold		Contig	
	**Length (bp)**	**Number**	**Length (bp)**	**Number**
Max length	877,183		91,298	
N10	244,122	385	19,843	3,963
N20	176,768	1,007	14,350	10,171
N30	136,595	1,823	11,014	18,435
N40	104,922	2,881	8,603	29,087
N50	80,559	4,262	6,641	42,800
N60	59,509	6,092	5,024	60,690
N70	40,171	8,668	3,589	84,991
N80	22,185	12,879	2,267	121,038
N90	7,287	22,166	1,085	186,167
Total_length	1,266,461,387		1,034,236,810	
number>=200 bp		152,316		399,107
number>=2000 bp		35,958		131,334
GC ratio	0.283		0.344	

Supplementary Table 19. Statistics of annotation results from various prediction methods

Gene set		Protein coding gene number	Average gene length (bp)	Average CDS length (bp)	Average exon per gene	Average exon length (bp)	Average intron length (bp)
De novo	AUGUSTUS[13]	59,666	6528.212	722.50	3.12	231.56	2738.46
	Glimmer[14]	47,655	16276.84	647.25	4.12	157.28	5017.17
Homologue	*A. thaliana*	24,593	6369.03	814.76	3.51	232.20	2213.81
	O. sativa	28,882	5892.98	781.940	3.25	240.49	2270.06
	P. equestris	41,596	4580.33	682.28	2.84	240.34	2119.88
	S. bicolor	26,286	6001.77	788.92	3.42	230.64	2153.53
	Z. mays	26,959	5701.55	764.15	3.30	231.56	2146.65
RNA-seq		24,626	12326.16	1114.50	4.40	253.05	2443.94
MAKER		30,791	10254.91	989.99	4.25	232.98	2628.63
Final set		28,910	10192.33	1002.25	4.13	242.77	2575.18

Supplementary Table 20. Statistics for gene function assignments from different databases

		Number	Percent (%)
Total		28,910	
Annotated	InterPro	18,700	64.68
	GO	13,436	46.48
	KEGG	14,826	51.28
	SwissProt	15,730	54.41
	TrEMBL	24,040	83.15
Unannotated		4,791	16.57

Supplementary Table 21. Enriched KEGG pathways for *D. catenatum* specific gene families

Map ID	Map Title	*P*-value	Adjusted *P*-value
map00350	Tyrosine metabolism	1.98E-09	1.23E-07
map00071	Fatty acid metabolism	1.12E-08	6.93E-07
map00010	Glycolysis / Gluconeogenesis	4.10E-05	0.002542
map00563	Glycosylphosphatidylinositol (GPI)-anchor biosynthesis	0.000236	0.014633

Supplementary Table 22. GO term enrichment results for Orchidaceae-specific gene families.

GO_ID	GO_Term	Class	*P*-value	Adjusted *P*-value
GO:0003682	Chromatin binding	MF	1.08E-07	6.96E-05
GO:0006355	Regulation of transcription, DNA-dependent	BP	4.77E-06	0.003067
GO:0010468	Regulation of gene expression	BP	8.86E-06	0.005698
GO:0019219	Regulation of nucleobase-containing compound metabolic process	BP	1.43E-05	0.009205
GO:0003700	Sequence-specific DNA binding transcription factor activity	MF	3.07E-05	0.019718
GO:0003677	DNA binding	MF	5.50E-05	0.035343

Supplementary Table 23. KEGG pathway enrichment results for Orchidaceae-specific gene families

Map ID	Map Title	*P*-value	Adjusted *P*-value
map00563	Glycosylphosphatidylinositol (GPI)-anchor biosynthesis	0.000242	0.020111

Supplementary Table 24. GO term enrichment results for monocot-specific gene families

GO ID	GO Term	Class	*P*-value	Adjusted *P*-value
GO:0043531	ADP binding	MF	6.35E-17	6.61E-15
GO:0006952	Defence response	BP	2.05E-15	2.13E-13
GO:0050896	Response to stimulus	BP	2.99E-11	3.11E-09
GO:0015299	Solute:hydrogen antiporter activity	MF	1.93E-05	0.002005
GO:0000156	Two-component response regulator activity	MF	3.31E-05	0.003442
GO:0000160	Two-component signal transduction system (phosphorelay)	BP	7.74E-05	0.008047

Supplementary Table 25. KEGG pathway enrichment results for monocot-specific gene families

Map ID	Map Title	*P*-value	Adjusted *P*-value
map03020	RNA polymerase	1.51E-12	1.51E-11
map00240	Pyrimidine metabolism	1.05E-10	1.05E-09
map00230	Purine metabolism	2.56E-10	2.56E-09
map00941	Flavonoid biosynthesis	0.001048	0.010484
map00945	Stilbenoid, diarylheptanoid and gingerol biosynthesis	0.00191	0.019102

Supplementary Table 26. The sequences of konjac EST clones used in the *CslA* and *CslD* phylogenetic trees

CslA	*AkEST1*	TFFHSDTFLFLTISHHHLPEQVCRPAGPPMLLIAICFVRAWQLIFNLEVTDVDELPALEAGSDG<br>QIHVLHGGPILPPSRLIQRRDPPHSGRSVEAKEGVGGGAYFLLHGEVVVQGHFLYPSHQALV<br>RVHEPPAGLDEGDVGVEGEEGDGAPEEVGLRLEVGVEDGHVVAVPDVAALHPLLEGARLVP<br>LPVVPDLVLDVDPLARPPLALHLHQVLDNGVGGVIKDLNYDAIGRPGQPTCSANGELVHLL<br>LVVRSGIWTRTMGYAELPISTSSLMGSHLVPPLRPPAKELDEEDDDAHVHTLHEQHQRHGQA<br>QHHGTRSRGTMTGARTCCHTSPICPARSSRPPLGMPPAAGSSTRGAPSCTAPHARRRPTTGPE<br>PAGLRLLCLSCVRFRPPPLFLILVRFSSVLHLSFYVRKNS
	AkEST2	RRKGSRSCRCGEGLCHLLASSSFGKIIAHIVTFIFYCVVIPITVFVPEVEIPKWGAIYIPSVITLL<br>NAVGTPRSIHLLVFWILFENVMSVHRTKATFIGLLEAGRVNEWVVTEKLGDALKAKAAAAA<br>ASVNNNKASKKPPPRFRIGDRLHVLELGVGAFLFFCACYDVAFGKNHFFIYLFLQASFAFVV<br>VWCRESVDXXRSAXSXRDAYYEYVVRRRRTTYT
	AkEST3	LPDLVLDVDPLARPPLALHLHQVLDNGVGGVIKDLNYDAIGRPGQPTCSANGELVHLLLVV<br>HGGFGRGPVGYAELPISTSSLMGSHLYRLSGRRPKSLTRSDDDAHRTHATDEQHQRHRTRH<br>STTATRSEGTMTGARTCCHTSPISPGHGSSRPPSELASGQPGSSTERRSILHRSPRPSPTHHRSR<br>TRGLPPPLFILCSFSAPLPCF
CslD	*AkEST1*	TGSLAVPREPLDAAIVAEAISVISCFYEDKTEWGRRVGWIYGSVTEDVVTGYRMHNRGWRS<br>VYCVTKRDAFRGTAPINLTDRLHQVLRWATGSVEIFFSRNNALFASRRMKFLQRVAYFNVGM<br>YPFTSIFLIVYCTLPAMSLFSGKFIVQSLSVMFLTFLLVITITLCLLAILEIRWSGITLHDWWRNE<br>QFWLIGGTSAHPAAVLQGLLKVIAGVDISFTLTSKPATDDNDDAFAELYVVKWSFLMVPPITI<br>MMINMIAIAVGVARTS
	AkEST2	KRHCTMASNNALKTSRSARLASSPSSLSASDVRPSVAGPLRPTVTFGRRTSSGRYVSYSRDDL<br>DSELGSGEFASYHVHIPATPDNQPAETAPVDTSISARVEEQYVSNSLFTGGFNSVTRAHLMDK<br>VIESEASHPQMAGAKGSSCAIPGCGARVMSDERGNDILPCECDFKICAECFADAVKGGEGVC<br>PGCKEPYKSTDMDEVVNNAGRPALSLPPPPAGMTKMERRLSLMRSAKLTRSQTGDFDHNR<br>WLFETKGTYGYGNAFWPKENGGGSDGGSSSGNGQPSELMSKPWRPLTRKLKIPAAILSPYR<br>LLIFVRMAALGLFLAWRIKHKNEDAIWLWGMSVVCEVWFAFSWLLDQLPKLCPINRATDLA<br>VLKEKFEAPGAHNPTGKSDLPGIDVFVSTADPEKEPPLVTANTILSILAADYPVEKLACYVSD<br>DGGALLTFEAMAEAASFANTWVPFCRKHDIEPRNPESYFSLKKDPYKNKLRPDFVKDRRRV<br>KREYDEFKVRINGLPDSIRRRSDAYHAREEIKAMKLQRETAGDEPLESVKIPKATWMADGTH<br>WPGTWTIPSAEHSRGDHAGIIQVMLKPPSDVPLHGDSDEARLLDLSDVDIRLPMLVYVSREK<br>RPGYDHNKKAGAMNALVRASAIMSNGPFILNLDCDHYIYNSQALREGMCFMMDRGGDRIC<br>YVQFPQRFEGIDPSDRYANNNTVFFDVNMRALDGLQGPVYVGTGCLFRRIALYGFDPPRSKD<br>HSPGCCSCCFPRSRKGLVAXXXXXXXXXXXXXGDELINISQEIWKLKHAH
	AkEST3	QFWLIGGTSAHLAAVLQGLLKVIAGIEISFTLTSKSAGDDVDDEFADLYVVKWTSLMIPPITIIF<br>VNIIAIAVGFSRTIYSELPQWSRLLGGVXXXXVLAHLYPFAKGLMGRRGRTPTIVFVWSGLIAI<br>TISLLWVAIKPPSGASQIGGSFTFP

Supplementary References

1. Wu, Z., Raven, P. H. & Hong, D. ed. *Flora of China* **25**, *Orchidaceae* (Beijing: Science Press & St. Louis: Missouri Botanical Garden Press. 2009).
2. Liu, Z. J. et al. Recent developments in the study of rapid propagation of *Dendrobium catenatum* Lindl. with a discussion on its scientific and Chinese names. *Plant Sci. J.* **29** (6): 763–772 (2011).
3. Wood, H. P. *The Dendrobiums* (A. R. G. Gantner Verlag Ruggell/Liechtenstein. 2006).
4. Kapitonov, V. V. & Jurka, J. A universal classification of eukaryotic transposable elements implemented in Repbase. *Nat. Rev. Genet.* **9**, 411–412; author reply 414 (2008).
5. Holt, C. & Yandell, M. MAKER2: an annotation pipeline and genome-database management tool for second-generation genome projects. *BMC Bioinformatics* **12**, 491 (2011).
6. Parra, G., Bradnam, K. & Korf, I. CEGMA: a pipeline to accurately annotate core genes in eukaryotic genomes. *Bioinformatics* **23**, 1061-1067 (2007).
7. Guindon, s. et al. New algorithms and methods to estimate maximum-likelihood phylogenies: assessing the performance of PhyML 3.0. *Syst. Biol.* **58**, 307–321 (2010).
8. Gaut, B. S., Morton, B. R., McCaig, B. C. & Clegg, M. T. Substitution rate comparisons between grasses and palms: synonymous rate differences at the nuclear gene *Adh* parallel rate differences at the plastid gene *rbc*L. *Proc. Natl Acad. Sci. USA* **93**, 10274–10279 (1996).
9. Maere, S. *et al.* Modeling gene and genome duplications in eukaryotes. *Proc. Natl Acad. Sci. USA* **102**, 5454–5459 (2005).
10. Gille, S. *et al.* Deep sequencing of voodoo lily (*Amorphophallus konjac*): an approach to identify relevant genes involved in the synthesis of the hemicellulose glucomannan. *Planta* **234**, 515–526 (2011).

11. Reyes, F. & Orellana, A. Golgi transporters: opening the gate to cell wall polysaccharide biosynthesis. *Curr. Opin. Plant Biol.* **11**, 244–251 (2008).

12. Xing, X. *et al*. A review of isolation process, structural characteristics, and bioactivities of water-soluble polysaccharides from *Dendrobium* plants. *Bioactive Carbohydrates and Dietary Fibre* **1**, 131–147 (2013).

13. Katsuraya, K.*et al*. Constitution of konjac glucomannan: chemical analysis and ^{13}C NMR spectroscopy, *Carbohydr. Polym.* **53**, 183–189 (2003).

14. Wang, J.H., Zha, X.Q., Luo, J.P. & Yang, X.F. An acetylated galactomannoglucan from the stems of *Dendrobium nobile* Lindl. *Carbohydr. Res.* **345**, 1023–1027 (2010).

15. Vieira, M.C. & Gil A.M. A solid state NMR study of locust bean gum galactomannan and Konjac glucomannan gels. *Carbohydr. Polym.* **60**, 439–448 (2005).

16. Rennie E.A & Scheller H.V. Xylan biosynthesis. *Curr. Opin. Biotechnol.* **26**, 100–107 (2014).

17. Hsieh, Y.S. *et al.* Structure and bioactivity of the polysaccharides in medicinal plant *Dendrobium huoshanense*. *Bioorgan. Med. Chem.***16**, 6054–6058 (2008).

18. Wang, Y., Mortimer, J.C., Davis, J., Dupree, P. & Keegstra, K. Identification of an additional protein involved in mannan biosynthesis. *Plant J.* **73**, 105–117 (2013).

19. Cribb P.J. *The Genus Paphiopedilum, 2nd edn*. Natural History Publications, Kota Kinabalu and Kew (1998).

20. URL: http://www.photoshop.com/

21. Kilian, J. *et al.* The AtGenExpress global stress expression data set: protocols, evaluation and model data analysis of UV-B light, drought and cold stress responses. *Plant Journal* **50 (2)**, 347–363 (2007).

22. Winter, D. *et al.* An "Electronic Fluorescent Pictograph" browser for exploring and analyzing large-scale biological data sets. *PLoS ONE* **2(8)**, e718 (2007).

23. Jain, M. *et al.* F-box proteins in rice. Genome-wide analysis, classification,

temporal and spatial gene expression during panicle and seed development, and regulation by light and abiotic stress. *Plant Physiology* **143(4)**, 1467–1483 (2007).

24. Rautengarten, C. *et al.* The Golgi localized bifunctional UDP-rhamnose/UDP-galactose transporter family of Arabidopsis. *Proc. Natl Acad. Sci. USA* **111(31)**, 11563–11568 (2014).
